# Apigeninidin-rich *Sorghum bicolor* (L. Moench) extracts suppress A549 cells proliferation and ameliorate toxicity of aflatoxin B1-mediated liver and kidney derangement in rats

**DOI:** 10.1038/s41598-022-10926-1

**Published:** 2022-05-06

**Authors:** Solomon E. Owumi, Abisola I. Kazeem, Bocheng Wu, Lucia O. Ishokare, Uche O. Arunsi, Adegboyega K. Oyelere

**Affiliations:** 1 Cancer Research and Molecular Biology Laboratories, University of Ibadan, Ibadan, Nigeria; 2 Nutrition and Industrial Biochemistry Laboratories, Department of Biochemistry, Faculty of Basic Medical Sciences, University of Ibadan, Ibadan, Nigeria; 3 School of Chemistry & Biochemistry, Georgia Institute of Technology, Atlanta, GA 30332-0400 USA; 4 Department of Cancer Immunology and Biotechnology, School of Medicine, University of Nottingham, Nottingham, NG7 2RD UK; 5 Parker H. Petit Institute for Bioengineering and Bioscience, Georgia Institute of Technology, Atlanta, GA 30332-0400 USA

**Keywords:** Drug discovery, Drug safety, Drug screening, Pharmacology, Toxicology

## Abstract

*Sorghum bicolor* plant has a high abundance of 3-deoxyanthocyanins, flavonoids and other polyphenol compounds that have been shown to offer numerous health benefits. Epidemiological studies have linked increased intake of *S. bicolor* to reduced risk of certain cancer types, including lung adenocarcinoma. *S. bicolor* extracts have shown beneficial effects in managing hepatorenal injuries. This study investigated the cytotoxic potential of three apigeninidin-rich extracts of *S. bicolor* (SBE-05, SBE-06 and SBE-07) against selected cancer cell lines and their ameliorative effect on aflatoxin B_1_ (AFB_1_)-mediated hepatorenal derangements in rats. We observed that, among the three potent extracts, SBE-06 more potently and selectively suppressed the growth of lung adenocarcinoma cell line (A549) (IC_50_ = 6.5 μg/mL). SBE-06 suppressed the expression of STAT3 but increased the expression of caspase 3. In addition, SBE-05, SBE-06 and SBE-07 inhibited oxidative and nitrosative stress, inflammation, and apoptosis and preserved the histoarchitectural networks of the liver and kidney of rats treated with AFB_1_. These in vitro and in vivo studies indicate the potential of these cheap and readily accessible extracts for cancer therapy and as chemo-preventive agents in preventing aflatoxin-related health issues.

## Introduction

Aflatoxin B_1_ (AFB_1_) is the most toxic among a group of mycotoxins contaminants in several food crops, especially grains such as corns, millet and sorghum, in the staple diets of developing countries. The production of Aflatoxins by *Aspergillus flavus* and *Aspergillus parasiticus* is favourable under high temperatures (between 24 and 35 °C) and high humidity (7–10%). These conditions are ambient in tropical and subtropical regions, namely Sub-Saharan Africa and Southeast Asia, that often experience a high incidence of aflatoxin contamination. Inability to store harvested crops in dry and temperature-controlled environments in developing countries increases contamination risks. Recognising the prevalence of food storage problems in these regions of the world, AFB_1_ is considered an unavoidable food contaminant by the US Food and Drug Administration (FDA)^1^.

Ingestion is the most common route of exposure to AFB_1_. Upon consumption of AFB_1_-contaminated meal, AFB_1_ is metabolised in the liver by microsomal mixed-function oxidase (MFO), a member of the cytochrome P450 (CYP) superfamily to different metabolites. Several isoforms of CYP mediate the bioactivation of AFB_1_ into toxic metabolites in different animals. Specifically, CYP1A1, CYP12A, CYP3A4, CYP2A13, CYP3A5, CYP3A37, and CYP2A5/2A6 orchestrate the bioactivation of AFB_1_ into AFBO, which is the most potent metabolite; CYP1A2 converts AFB_1_ to aflatoxin M1 (AFM1), CYP1A and CYP3A mediate the biotransformation of AFB_1_ into aflatoxicol, while CYP1A2, CYP1A5, CYP3A4 and CYP3A37 drive the bioactivation of AFB_1_ into aflatoxin Q1 (AFQ1). Furthermore, the toxic metabolite, AFBO, is conjugated to glutathione to form aflatoxin B_1_ glutathione conjugate (AFB_1_-GSH) in a phase-2 reaction step catalysed by glutathione S-transferase^2–4^. AFB_1_-GSH, an inert metabolite, is converted in a sequence of reaction steps coordinated by γ-glutamyl transpeptidase (GGT), dipeptidase (DPEP) and N-acetyltransferase (NAT) into aflatoxin B_1_ mercapturic acid adduct—which is excreted in the urine^5^. Suppression of the activity of GST and other phase-2 reaction enzymes causes the build-up of AFBO in the liver and kidney of lactating goats and rats^6^. AFBO, through its reaction with biomolecules such as DNA, RNA, proteins, and lipids, is responsible for the toxic, carcinogenic and teratogenic effects of AFB_1_^7–9^. The harmful biochemical and physiological outcomes are due to the capability of AFBO to inhibit proteins, RNA, and DNA synthesis^10–13^ while triggering lipid peroxidation, oxidative DNA damage, and the formation of protein–protein crosslinks and DNA adducts^14–16^.

AFB_1_ has been shown to cause malnutrition and growth impairment in humans and animals^17^. In rats, there is ample evidence demonstrating the toxicities of AFB_1_ to the hepatorenal system. Previous findings revealed that the ingestion of AFB_1_ altered the natural intracellular antioxidant/pro-oxidant balance and anti-inflammatory/pro-inflammatory rheostat in favour of damaging pro-oxidant and pro-inflammatory states, respectively, occasioning altered mean liver and kidney weights, marked increase in reactive oxygen species (ROS) and reactive nitrogen species (RNS), pro-inflammatory molecules as well as significant diminutions of endogenous antioxidants and anti-inflammatory cytokine levels in the liver and kidney tissues^18–20^. Additionally, histoarchitectural assessment of the toxic effects of AFB_1_ on rats reveals severe vacuolar and hydropic degeneration, enlarged nuclei, nuclear inclusion, and dysplastic changes in hepatocytes^21–23^. In contrast, hydropic and vacuolar degeneration, tubular degeneration, epithelial swelling and blabbing, infiltration of inflammatory cells, and segmental glomerular necrosis were observed in kidney tissues of rats^14,24^. Mechanistically, these changes are attributed to a marked increase in the expression of tumour necrosis factor-alpha (TNF-α), interleukin-6 (IL-6) and interleukin-1 beta (IL-1β), inducible nitric oxide synthase (iNOS), cyclooxygenase-2 (COX-2), nuclear factor κappa B (NF-κB), and inducible nitric oxide synthase (iNOS) signalling with concomitant suppression in the activity of NF-κB inhibitor (IκB). Collectively, these changes trigger inflammation^25,26^. Also observed are upregulation of kelch-like ECH-associated protein-1a (Keap1a), decrease in the expression of nuclear factor erythroid 2-related factor-2 (Nrf2) and heme oxygenase-1 (Ho-1) signalling^27,28^ with the resultant reduction of mRNA expression of GSH-Px, SOD, CAT, and GST^25,29^. These last changes contribute to the AFB_1_ induction of oxidative and nitrosative stress and increase Bax, Caspase-3 and fatty acid synthetase (FAS) mRNA expression. FAS-associated death domain (FADD), TNF-associated death domain (TRADD), and caspase-8 with a simultaneous decrease in the level of Bcl-2, thus promoting apoptosis^30^ in the hepatorenal system.

**Scheme 1:**
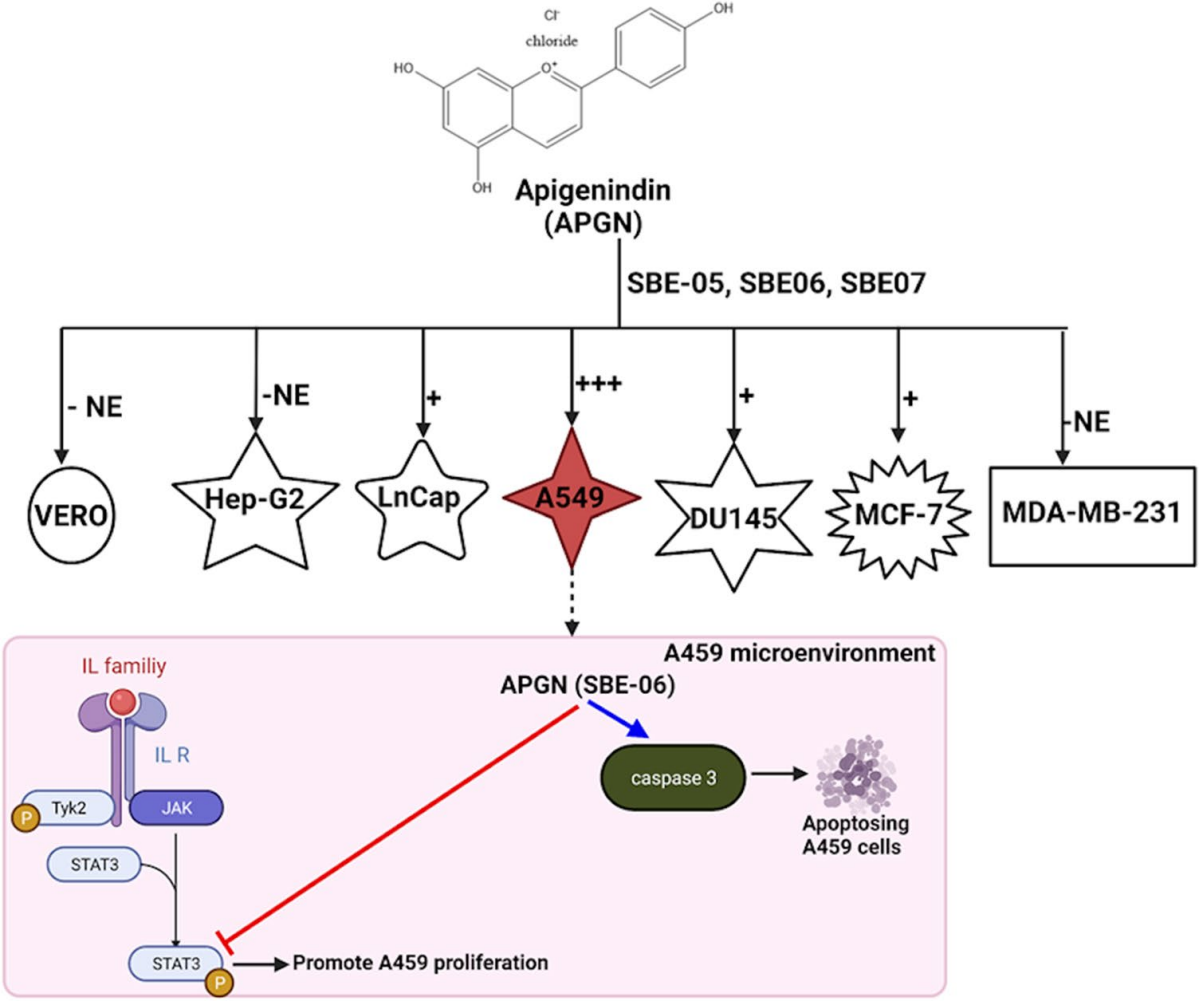
Proposed mechanism of SBE-06-mediated suppression of A549 cell growth. SBE-06 downregulated the expression of p-STAT3 but upregulated that of caspase 3 in A549 cells. p-STAT3: phosphorylated signal transducer and activator of transcription 3. Created by https://app.biorender.com/.

**Scheme 2:**
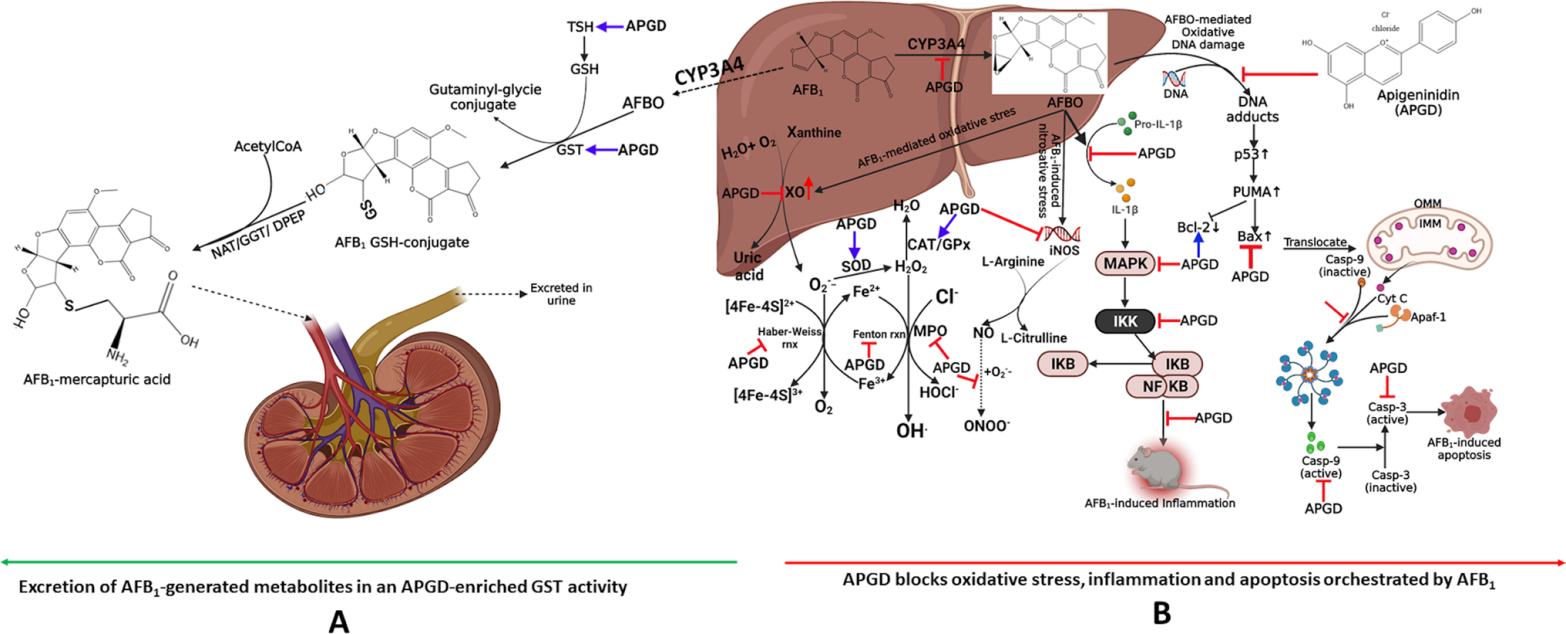
Proposed mechanism of SBE-05, SBE-06 and SBE-07 ameliorative effect on AFB_1_-mediated toxicities in the liver and kidney of an experimental rat model. SBE-05, SBE-06 and SBE-07 averted AFB_1_-induced oxidative and nitrosative stress, inflammation, and apoptosis by attenuating the activity of CYP450 isoforms, NF-kB-mediated generation of pro-inflammatory cytokines, IL-1β as well as altering the Bcl-2/Bax ratio in favour of the action of caspase 9 and caspase 3. Created by https://app.biorender.com/.

Paradoxically, some of the grains contaminated with AFB_1_ and other plants contain protective phytochemicals that could be potentially used to reduce AFB_1_ poisoning. Extracts from maize^31^, *Piper beetle*^32^, and *Heracleum persicum*, *Peganum harmala*, and *Trachyspermum ammi*^33^, have been shown to inhibit AFB_1_ biosynthesis and *A. flavus* growth. *Sorghum bicolor* plant has a high abundance of 3-deoxyanthocyanins, flavonoids and other polyphenol compounds that elicit phytoalexin activity^34–36^, and it is less susceptible to aflatoxin contamination in the field^37,38^. However, sorghum grains are frequently contaminated with aflatoxins at higher levels under storage^39,40^. It is plausible that the depletion of these protective phytochemicals, in addition to poor storage conditions, creates conditions conducive for the growth of *Aspergillus*, hence contamination. Due to the anti-inflammatory and anti-oxidative stress effects of the 3-deoxyanthocyanins, flavonoids and other polyphenol compounds found in sorghum^41^, it is conceivable that some of these phytochemicals could mitigate the toxic effects of AFB_1_. Given this possibility, the goal of this study is to investigate the effects of 3-deoxyanthocyanins-rich extracts of *S. bicolor* (apigeninidin) on the toxic effects of AFB_1_ exposure on the hepatorenal system of rats as well as assess its cytotoxic potential against different cell lines. To elucidate this, we extracted bioactive compounds in *S. bicolor*. Using liquid chromatography-mass spectrometry (LC–MS) and high-resolution mass spectrometry (HR-MS), these extracts were identified as apigeninidin. The extracted fractions were assessed for their in-vitro cytotoxic potential against selected cancer cell lines. Additionally, the extracts were evaluated for their ameliorative tendency against the AFB_1_-induced hepatorenal toxicity model in male Wistar rats. Finally, we proposed a plausible mechanism highlighting the anticancer effect of the most active fraction against A549 cells and the protective effect of apigeninidin-rich extracts against AFB_1_-induced oxidative and nitrosative stress, inflammation, and apoptosis in rats (Scheme 1 and 2).

## Materials and methods

### Chemicals, reagents and kits

The materials used for this study are listed in Table 1. All other reagents and chemicals used were obtained commercially and are of analytical grade.

**Table 1:** List of chemicals, reagents and kits used for the estimation of the oxidative, inflammatory, and apoptotic biomarkers in the liver and kidney of rats.

Chemical name	Catalogue No	Company
1-Chloro-2,4-dinitrobenzene (CDNB)	97-00-7	Sigma-Aldrich Inc. (St Louis, MO, USA)
2’,7’-dichlorodihydrofluorescin diacetate	4091-99-0	Sigma-Aldrich Inc. (St Louis, MO, USA)
5,5’-dithiobis-(2-nitrobenzoic acid)	69-78-3	Sigma-Aldrich Inc. (St Louis, MO, USA)
Rat 8-OHdG ELISA kit	E-EL-0028	Elabscience Biotechnology Company (Wuhan, China)
Aflatoxin B1	1162-65-8	Sigma-Aldrich Inc. (St Louis, MO, USA)
Alanine aminotransferase (ALT)	AL7930	Randox™ Laboratories Limited, (Crumlin, UK)
Alkaline phosphatase (ALP)	AP3803	Randox™ Laboratories Limited, (Crumlin, UK)
Aspartate aminotransferase (AST)	AS101	Randox™ Laboratories Limited, (Crumlin, UK)
Rat Caspase -3 ELISA kit	E-EL-R0160	Elabscience Biotechnology Company (Wuhan, China)
Caspase-9 ELISA kit	E-EL-R0163	Elabscience Biotechnology Company (Wuhan, China)
Creatinine	CR510	Randox™ Laboratories Limited, (Crumlin, UK)
Dipotassium hydrogen phosphate trihydrate	7758-11-4	AK Scientific, Union City, USA
Epinephrine	51-43-4	Sigma-Aldrich Inc. (St Louis, MO, USA)
Folin–ciocalteau reagent	125,629	JT Baker (Phillipsburg, PH, USA)
Gamma-glutamyltransferase (ɣ-GT)	12,013	Randox™ Laboratories Limited, (Crumlin, UK)
Hydrogen peroxide (H_2_O_2_)	7722-84-1	Sigma-Aldrich Inc. (St Louis, MO, USA)
Rat IL-10 (Interleukin -10) ELISA kit	E-EL-R0016	Elabscience Biotechnology Company (Wuhan, China)
Rat (IL-1β) Interleukin-1beta ELISA kit	E-EL-R0012	Elabscience Biotechnology Company (Wuhan, China)
N-(1-Naphthyl)ethylenediamine hydrochloride	1465-25-4	Avishkar Lab Tech Chemicals, India
O-Dianisidine	119-90-4	Sigma-Aldrich Inc. (St Louis, MO, USA)
Potassium Chloride	7447-40-7	AK Scientific, Union City, USA
Potassium dihydrogen phosphate	7778-77-0	AK Scientific, Union City, USA
Reduced glutathione (GSH)	70-18-8	Sigma-Aldrich Inc. (St Louis, MO, USA)
Sodium carbonate anhydrous	497-19-8	Loba Chemie, Mumbai, India
Sodium Carboxymethyl cellulose	9004-32-4	Sigma-Aldrich Inc. (St Louis, MO, USA)
Sodium hydrogen carbonate	144-55-8	Loba Chemie, Mumbai, India
Sodium hydroxide pellets	1310-73-2	Molychem, Mumbai India
Sodium–Potassium tartrate	6381-59-5	Sigma-Aldrich Inc. (St Louis, MO, USA)
Sulphosalicylic acid	5965-83-3	Sigma-Aldrich Inc. (St Louis, MO, USA)
Thiobarbituric acid	504-17-6	AK Scientific, Union City, USA
Thiobarbituric acid (TBA)	504-17-6	Sigma-Aldrich Inc. (St Louis, MO, USA)
Trichloroacetic acid	76-03-9	Molychem, Mumbai India
Urea	10,505	Randox™ Laboratories Limited, (Crumlin, UK)

### Collection, identification, and processing of plant sample

**Figure 1:**
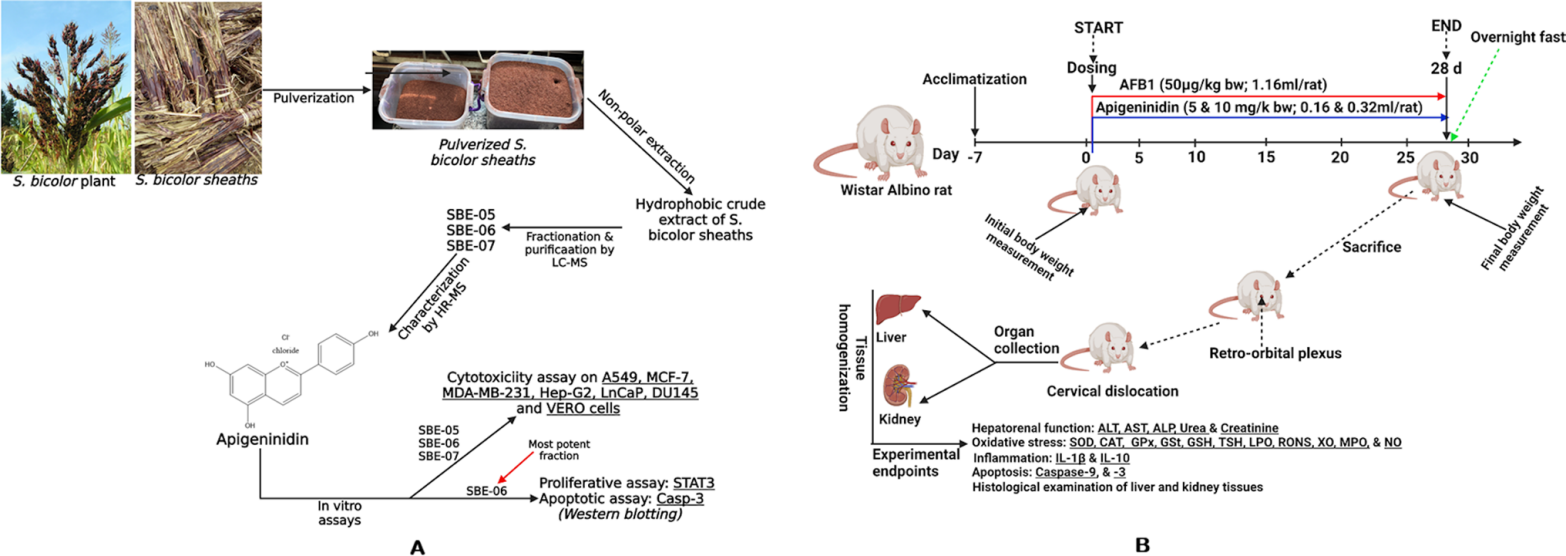
Experimental protocol of AFB_1_ and Apigeninidin-rich fractions of *S. bicolor* extracts, (**A**): Extraction, fractionation, purification and characterisation of *S. bicolor* sheaths, and in vitro anticancer screening of Apigeninidin-rich fractions of *S. bicolor* extracts, (**B**): In vivo screening of the hepatoprotective effect of SBE-05, SBE-06 and SBE-07 on AFB1-challenged adult male Wistar Albino rats for 28 consecutive days. Created by https://app.biorender.com/.

The dried *Sorghum bicolor* sheaths (5 kg) (Fig. 1A) were purchased from the Bodija market in Ibadan North Local Government Area of Oyo State, Nigeria. Geographically, the Bodija market lies between longitude 3 54′36’’E and 3 55′ 12’’E and latitude 7 25′ 52’’N and 7 26′ 22’’N^42^. The plant samples were transported in a polythene bag to the Herbarium of the Department of Botany, University of Ibadan, Ibadan, Nigeria, for identification by a veteran taxonomist Mr Donatus Esimekhuai. The sample of *S. bicolor* was identified and deposited in the Department of Botany, and a voucher specimen-Accession number: UIH-23118-was assigned for future reference. The experimental research on the plant—*S. bicolor*-complied with all relevant institutional, national, and international guidelines and legislation. The plant was sorted to remove dirt and other extraneous materials and pulverised into fine powder.

### Extraction and phytochemical characterisation of *S. bicolor*

The pulverised *S. bicolor* (120 g) is degreased with CH_2_Cl_2_ and subsequently extracted with CH_2_Cl_2_: MeOH 10:1 (twice) at 60 °C and 0.1% HCl in EtOH at room temperature. Each filtrate was evaporated off to give SBE-05 (2.6 g), SBE-06 (1.44 g) and SBE-07 (4.85 g) as brownish-red solid.

### LC–MS analysis

The isolated solid from each fraction—SBE-05, SBE-06 and SBE-07—was analysed by LC–MS, monitoring at 420 nm, on a Bruker amaZon SL ion trap mass spectrometer coupled to an Agilent 1260 HPLC. Chromatography was performed on Phenomenex C18 reversed-phase HPLC column (250 × 4.6 mm; S/NO: H17-238,591) at a flow rate of 0.5 mL/min; using 0.1% v/v formic acid in H_2_O (solvent A) and 0.1% v/v formic acid in MeCN (solvent B). The solvent gradient for chromatography elution is as follows: 0–5 min at 5% solvent B; 5–25 min from 5 to 100% solvent B; 25–28 min at 100% solvent B; 28–29 min from 100 to 5% solvent B; and 29–32 min at 5% solvent B. Mass spectrometry data were collected in the positive and negative ionisation modes in the mass range *m/z* 100–1000 Da.

### In vitro cell cytotoxicity assay

A549 cells were maintained in Dulbecco’s Modified Eagle Medium (DMEM) (Corning, 10-017-CV), supplemented with 10% fetal bovine serum (FBS) (Corning, 35-010-CV). Cells were seeded into a 96-well plate (2000–4000 cells/well) for 24 h before treatment. Subsequently, cells were treated with various concentrations of SBE-06 in 1% DMSO solution of medium for another 72 h. The effect of SBE-06 on cell viability was measured using the MTS assay (CellTiter 96 Aqueous One Solution, Promega, Madison, WI) as described by the manufacturer. The cytotoxicity IC_50_s were determined using Prism GraphPad 8.

### Western blot

A549 cells were seeded into a 6-well plate at 1*10^6^/well in DMEM for 24 h. Then, 5 and 10 µg/mL SBE-06 solutions in DMSO and DMSO were added to the cell culture media such that the final DMSO level is 0.1%. Cells were treated for 24 h, and the medium and cells were scraped from the plate to Eppendorf tubes. The cells in the medium were centrifuged at rpm = 7 for 5 min. Then, the supernatants were discarded, and the pellet was re-suspended with 1 mL of 1 × cold PBS. The cells were centrifuged again at the same setting. The PBS was discarded, and the cells were lysed with RIPA buffer (110μL) (VWR, VWRVN653-100ML) buffer containing phosphatase inhibitor (Fisher Thermo, A32957) and protease inhibitor (Fisher Thermo, A32955). The lysate was vortexed for 30 s, then sonication for 90 s in a water bath. The lysate was then centrifuged at 14,000 rpm for 15 min, and the supernatant was collected and transferred. The total protein concentration was determined using a BCA protein assay kit (BioVision, K813-2500). Based on the results from the BSA assay, the lysates were diluted to make equal protein concentration, and 20–40μg of each lysate was loaded to each well on a TGX MIDI 4–20% gel (Bio-Rad, cat. 5,671,093) and ran at 150 V for 70 min. Subsequently, the gel was transferred to the Turbo PDVF membrane (Bio-Rad, 1,704,273) after blocking with 5% dry milk for 1 h. The membrane was washed three times with TBS-T buffer, and the membrane was incubated overnight with AR (Santa Cruz, sc-7305), p-STAT3/STAT3 (Cell signalling, D3A7/D1B2J), GAPDH (MA116757, Thermo Fisher), and Caspase 3 (Cell signalling, 9662 s/9664 s) antibodies. The membrane was washed with TBS-T for 3 × 5 min on the second day. A secondary antibody (Immunoreagents, part. IR2173) was added, and the membrane was incubated with agitation for one hour. Bands were quantified using the Odyssey CLx Image system.

### Animal welfare, sample size calculation and experimental design

The study was conducted in line with the 3R’s guidelines, including Replacement, Reduction, and Refinement for the welfare and use of experimental animals^43,44^. Based on these guidelines, we assess the cytotoxic potentials of the apigeninidin-rich fractions in different cancer cell lines. Also, the ameliorative effect of apigeninidin-rich fractions was examined in AFB_1_-challenged rats' hepatorenal system. The sample size for the study was estimated using G*Power Software version 3.1.9.4^45^ with an effect size of 0.4 at *p* < 0.05 for one way analysis of variance (ANOVA)^46^ to obtain a total samples size of 125 at 95% power. Out of the 125 animals obtained from our sample size estimation, 48 healthy male Wistar Albino rats, comprising eight cohorts of experimental animals (n = 6) and weighing approximately 164 ± 5 g body weight were purchased from the Experimental Animal Facility of the Faculty of Veterinary Medicine, University of Ibadan, Ibadan, Nigeria. Rats were housed in standard cages placed in an aerated animal house of the Department of Biochemistry, Faculty of Basic Medical Sciences, University of Ibadan, Ibadan, with a 12 h light–dark cycle. Rats were fed with standard rat pellets (purchased from Ladokun Feeds Limited, Ibadan, Nigeria) and water at will and allowed to acclimate for seven days before administration of AFB_1_ and SBE. The protocols for the care and use of experimental animals adopted in this study were carried out after the institutional approval was obtained (UI-ACUREC/032-0521-7) and are in conformity with the ratified rules of the University of Ibadan Ethical Committee and the US National Institute of Health. Rats in the eight cohorts of animals were subjected to 28 d successive treatments of AFB_1_ and SBE (Fig. 1B). The experimental dose of AFB1 (50 μg/kg) was adapted from our previous study^14,47^ while SBE05, SBE-06, and SBE-07 (5 and 10 mg/kg) were based on data from the cell assay. The gavage volumes taken from specific stock solutions of SBE (5 and 10 mg/kg) and AFB_1_ were 0.16, 0.32 and 0.16 mL, respectively prepared by dissolving 150 mg SBE in 30 mL of carboxymethyl cellulose (0.5%) and 5.5 mg AFB_1_ in 110 mL of corn oil. Briefly, rats in the control group received 0.32 mL of carboxymethyl cellulose (0.5%), AFB_1_ alone received 0.16 mL of AFB1 (50 μg/kg bw) per os (*p.o*), AFB_1_ + SBE-05-D_1_ received 0.16 mL of AFB_1_ (50 μg/kg bw) and 0.16 mL of SBE-05-D_1_ (5 mg/kg) *p.o*., AFB_1_ + SBE-05-D_2_ received 0.16 mL of AFB_1_ (50 μg/kg bw) and 0.32 mL of SBE-05-D_2_ (10 mg/kg) *p.o*., AFB_1_ + SBE-06-D_1_ received 0.16 mL of AFB_1_ (50 μg/kg bw) and 0.16 mL of SBE-06-D_1_ (5 mg/kg) *p.o*., AFB_1_ + SBE-06-D_2_ received 0.16 mL of AFB_1_ (50 μg/kg bw) and 0.32 mL of SBE-06-D_2_ (10 mg/kg) *p.o*., AFB_1_ + SBE-07-D_1_ received 0.16 mL of AFB_1_ (50 μg/kg bw) and 0.16 mL of SBE-07-D_1_ (5 mg/kg) *p.o*. and AFB_1_ + SBE-07-D_2_ received 0.16 mL of AFB_1_ (50 μg/kg bw) and 0.32 mL of SBE-07-D_2_ (10 mg/kg) *p.o*.

### Tissue and organ harvest and processing

At the expiration of the 28-day treatment, the rats from all cohorts fasted for 24 h. The whole blood was collected in non-heparinised tubes via retro-orbital venous plexus and allowed to clot. The rats were sacrificed by dislocating their cervical vertebrae after carbon dioxide (CO_2_) asphyxiation^48–50^. The clotted blood samples were centrifuged at 3000 rpm for 10 min. Organs such as the liver and kidney were carefully removed and prepared for biochemical and histological assays after measuring the organ’s weight with a USS-DBS16 Analytical Balance (Cleveland, OH, USA). The liver and kidneys of sacrificed rats were further excised into two portions processed for histological investigation and biochemical analyses, respectively. For the biochemical studies, the harvested organs were homogenised −2 g of the liver in 8 mL of homogenizing buffer (in 0.1 M phosphate buffer, pH 7.4) using a glass-Teflon homogeniser. The kidney homogenate was prepared by homogenising the left or right kidney (mean weight: 1.12 g) in 4 mL of the homogenising buffer. The homogenates were centrifuged at 12,000 rpm for 15 min with a cold Eppendorf 5417R centrifuge (Hamburg, Germany). The supernatants (mitochondrial fractions) were collected and frozen in aliquots before biochemical analyses.

### Estimation of hepatorenal function parameters

The activities of liver function enzymes-aspartate aminotransferase (AST), alanine aminotransferase (ALT), alkaline phosphatase (ALP); and kidney function—creatinine and urea were assayed. Using sera from each cohort of experimental animals and ready to use kits per the manufacturer’s directives as reported previously^51^.

### Evaluation of oxidative stress, inflammation, and apoptosis in the hepatorenal system

The frozen mitochondrial fractions of the liver and kidney tissues were used to measure the level of oxidative stress, inflammation and apoptosis in the liver and kidney of rats challenged with AFB_1_ and remedied with different SBE fractions. The total proteins of the liver and kidney tissues were estimated following an established protocol^52^ to determine the exact concentration of any protein of interest in the tissue. The markers of oxidative and nitrosative stress in the liver and kidney of rats were determined following well-documented protocols: Superoxide dismutase (SOD) activity^53,54^; catalase (CAT) activity^55,56^ using H_2_O_2_ as a substrate; glutathione S-transferase (GST) activity^57^; glutathione peroxidase (GPx) activity^58,59^; reduced glutathione (GSH) level^60,61^. Also, total sulfhydryl (TSH) level^62,63^; xanthine oxidase (XO) activity^63,64^; myeloperoxidase (MPO) activity^18,65^; nitric oxide (NO) level^48,66^ were also evaluated accordingly. The level of Malondialdehyde (MDA) and reactive oxygen and nitrogen species (RONS) was determined as previously described^67,68^. Also, biomarkers of inflammation, -IL-1β and IL-10 concentrations- apoptosis -caspase-9 and -3 activities- in the liver and kidney of rats were determined using rat specific enzyme-linked immunosorbent (ELISA) kits following the manufacturer’s protocol reported previously^69^. All readings were obtained using a Spectra Max™ 384 Molecular Devices plate reader (San Jose, CA, USA).

### Histopathological assessment of the liver and kidney tissues

Before the histopathological examination, each experimental rat's liver and kidney sections were fixed in 10% neutral buffered formalin. The tissues were prepared for histological assessment via a standard paraffin-wax embedded method of^70^ as adapted from^71^. Approximately 5 μm thickness of the liver and kidney sections were stained with haematoxylin and eosin, and the slides were coded before being probed with a Carl Zeiss Axio light microscope (Gottingen, Germany). After the examination, representative images taken with a Zeiss Axiocam 512 camera (Gottingen, Germany) attached to the microscope were analysed by a pathologist unaware of the various treatment cohorts from which the slides were prepared.

### Statistical analysis

Paired student t-tests were performed to analyse the data generated from this study to determine the significance level in the mean body weight of rats before and after treatment. In addition, a one-way analysis of variance (ANOVA) followed by a post-hoc test (Tukey) was used to identify significant differences across the different cohorts of rats using GraphPad Prism version 8.3.0 for Mac (www.graphpad.com; GraphPad, CA, USA,). The results are expressed as the mean ± SD of replicates, and statistically significant differences were set at a value of *p* < *0.05*.

### Ethical approval

All experiments were performed following relevant guidelines and regulations and adhere to the ARRIVE guidelines (https://arriveguidelines.org) to report animal experiments. The protocols for the care and use of experimental animals in this study were approved by the University of Ibadan, Animal Care and Use in Research Ethical Committee with approval number: UI-ACUREC/032–0521-7. The experimental research on *S. bicolor* complied with all relevant institutional, national, and international guidelines and legislation. The sample of *S. bicolor* was identified correctly and deposited in the University of Ibadan, Department of Botany and a voucher specimen -Accession number: UIH-23118- was assigned for future reference.

## Results

### Analysis of fractions from *S. bicolor*

**Figure 2:**
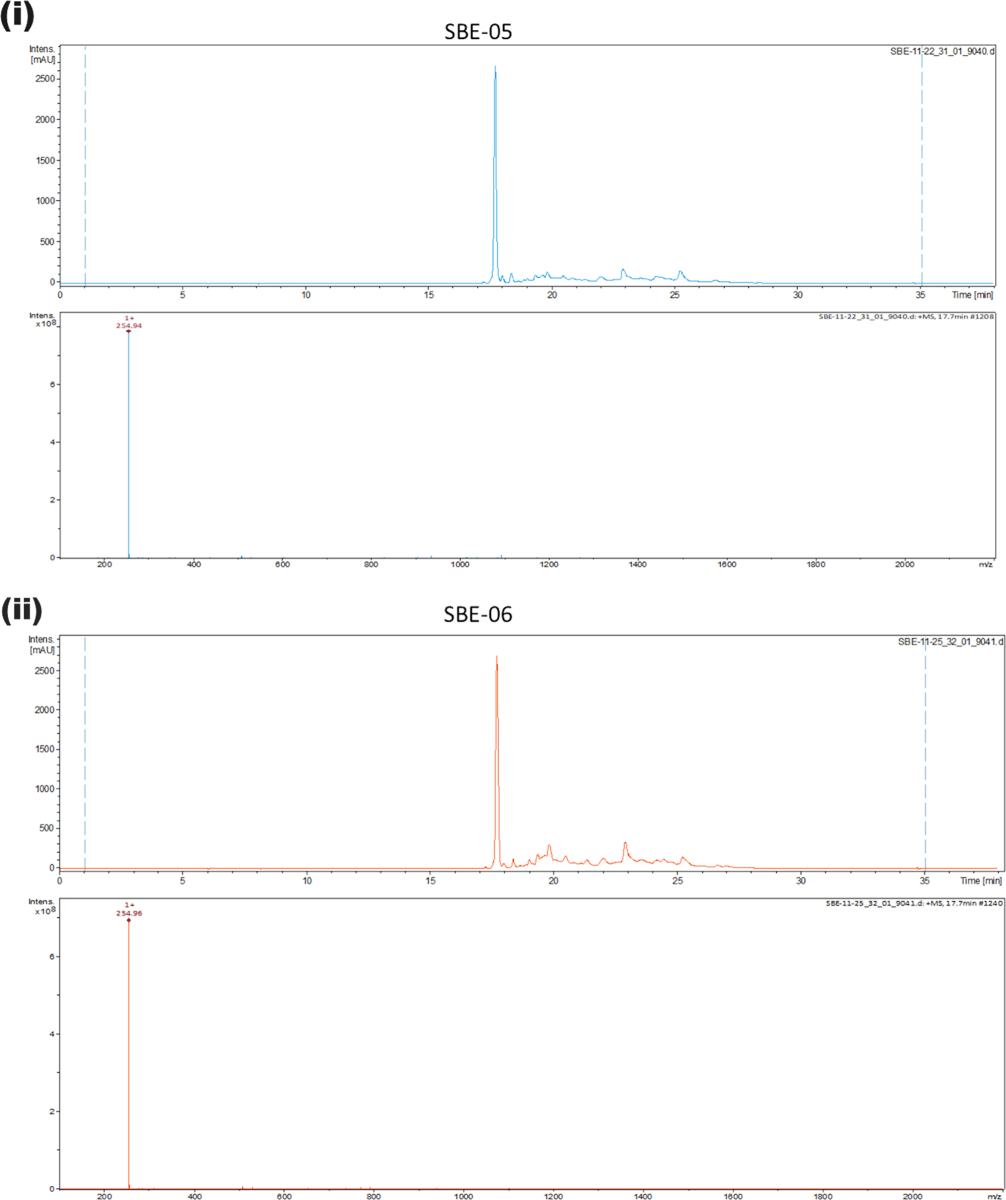

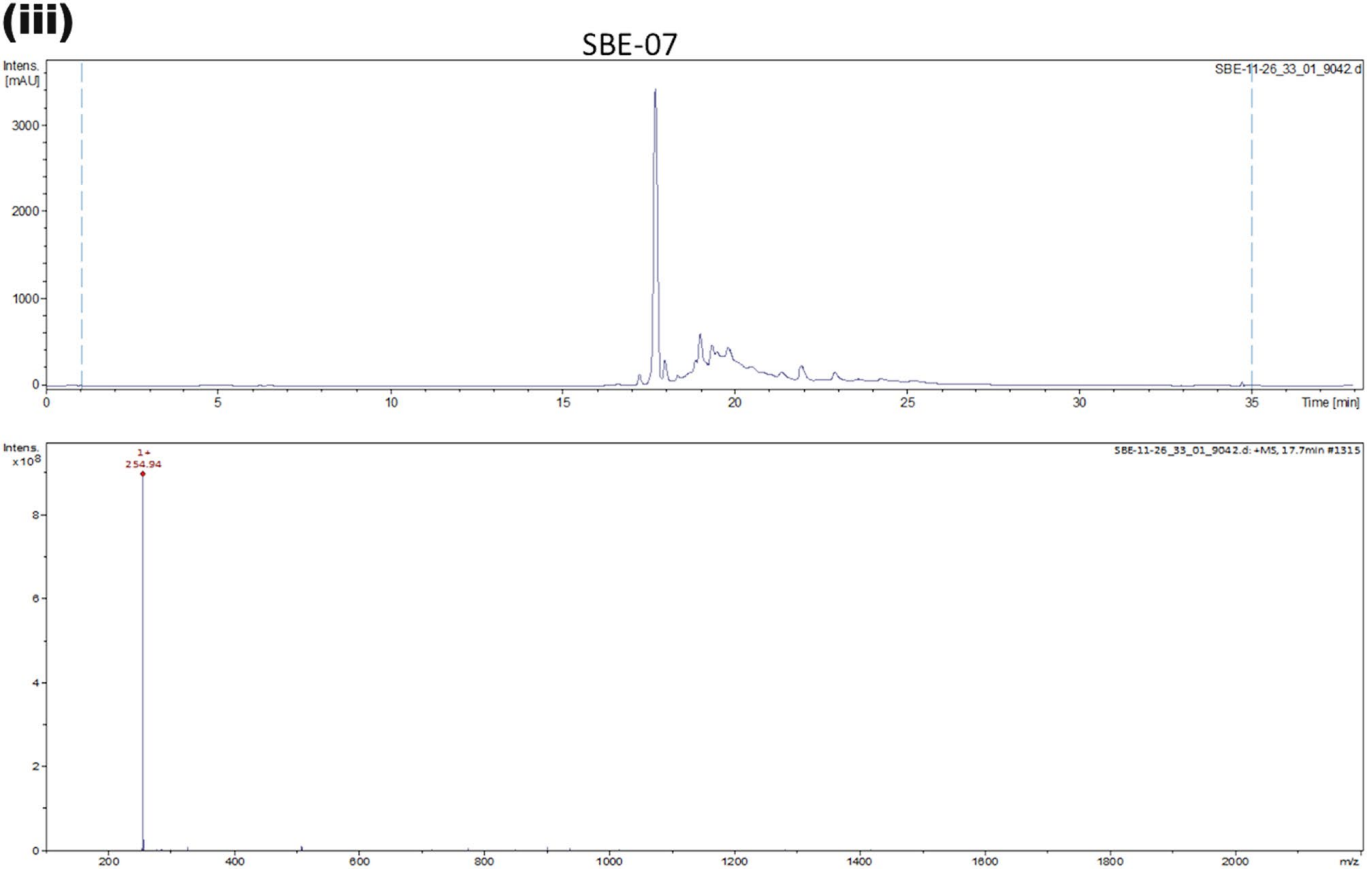
(i–iii). LC–MS analysis revealed enrichment of a compound with a molecular weight of 255 in SBE-05, SBE-06 and SBE-07*.* SBE: SBE: *Sorghum bicolor* extract.

The isolated solid from each fraction was analysed by LC–MS, monitoring at 420 nm. It was observed that each sample is enriched in a peak with a molecular weight of 255 (retention time = 17.7 min) with variation in the accompanying impurity profiles (Fig. 2i–iii). The identity of the enriched peak in each sample was confirmed by High-Resolution Mass Spectrometry to be apigeninidin (HRMS (ESI) Calcd for C_15_H_11_O_4_^+^, [M]^+^ 255.0562, found 255.0562), a 3-deoxyanthocyanin which is the primary pigment in of *S. bicolor*. Intriguingly, this treatment did not furnish significant amounts of other 3-deoxyanthocyanins and flavonoids found in *S. bicolor*^72^.

### In vitro study: evaluation of the cell cytotoxicity of SBE-05, SBE-06 and SBE-07

**Figure 3:**
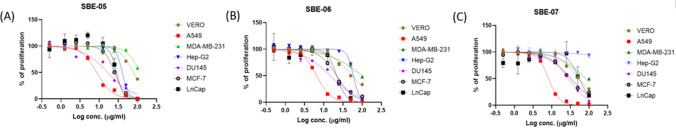
(**A**–**C**): Cytotoxic potential of SBE-05, SBE-06 and SBE-07 against A549, MCF-7, MDA-MB-231, Hep-G2, LnCaP, DU145 and VERO cells.

Apigeninidin is cytotoxic to some cancer cell lines^73^. To gauge the concentration range that may be tolerated by the test animals in in vivo study, we first evaluated the effects of SBE-05, SBE-06 and SBE-07 on the viability of selected normal and cancer cell lines. We investigated seven cell lines: VERO (African green monkey normal kidney epithelial cell), Hep-G2 (hepatocellular carcinoma), A549 (lung adenocarcinoma), MDA-MB-231 (triple-negative breast adenocarcinoma), MCF-7 (ER ( +) breast adenocarcinoma), LnCaP (AR ( +) prostate cancer) and DU145 (AR (-) prostate cancer) cell lines. Among the tested cancer cell lines, we observed that SBE-05, SBE-06 and SBE-07 have enhanced cytotoxicity against A549, with SBE-06 being slightly more potent with IC_50_ of 6.5 μg/mL (approximately 25.7 μM, assuming 100% apigeninidin). The IC_50_s of SBE-05 and SBE-07 against A549 are 9.4 and 8.3 μg/mL, respectively (Fig. 3 and Table 2). MDA-MB-231 and Hep-G2 cells are the least responsive to treating the cancer cell lines we investigated. Gratifyingly, SBE-05, SBE-06 and SBE-07 are less toxic to VERO, the normal representative cells (IC_50_ range of 55–60 μg/mL; equivalent to 60 mg/kg, assuming a density of 1 g/mL for the solid). The preceding findings suggest that a dose below 60 mg/kg may be suitable for in vivo evaluation.

**Table 2:** IC_50_ (ug/mL) of SBE-05, SBE-06 and SBE-07 against A549, MCF-7, MDA-MB-231, Hep-G2, LnCaP, DU145 and VERO cells extracted from the dose response curves.

Compound	IC50 (μg/mL)						
	A549	VERO	MDA-MB-231	MCF-7	LnCap	DU145	Hep-G2
SBE-05	9.4	59.5	> 100	25.2	28.5	26.4	40.3
SBE-06	6.5	54.7	99.9	21.6	21.7	14.6	58.9
SBE-07	8.3	55.7	81.9	33.0	55.3	28.5	> 100

*SBE* Sorghum Bicolor extract, *IC*_*50*_ Half maximal inhibitory concentration, *A549* Lung adenocarcinoma, *VERO* African green monkey normal kidney epithelial cell, *MDA-MB-231* Triple negative breast adenocarcinoma cell lines, *MCF-7* ER ( +) breast adenocarcinoma cell lines, *LnCap* AR ( +) prostate cancer cell lines, *DU145* AR (-) prostate cancer cell lines, *Hep-G2* Hepatocellular carcinoma cell lines.

**Figure 4:**
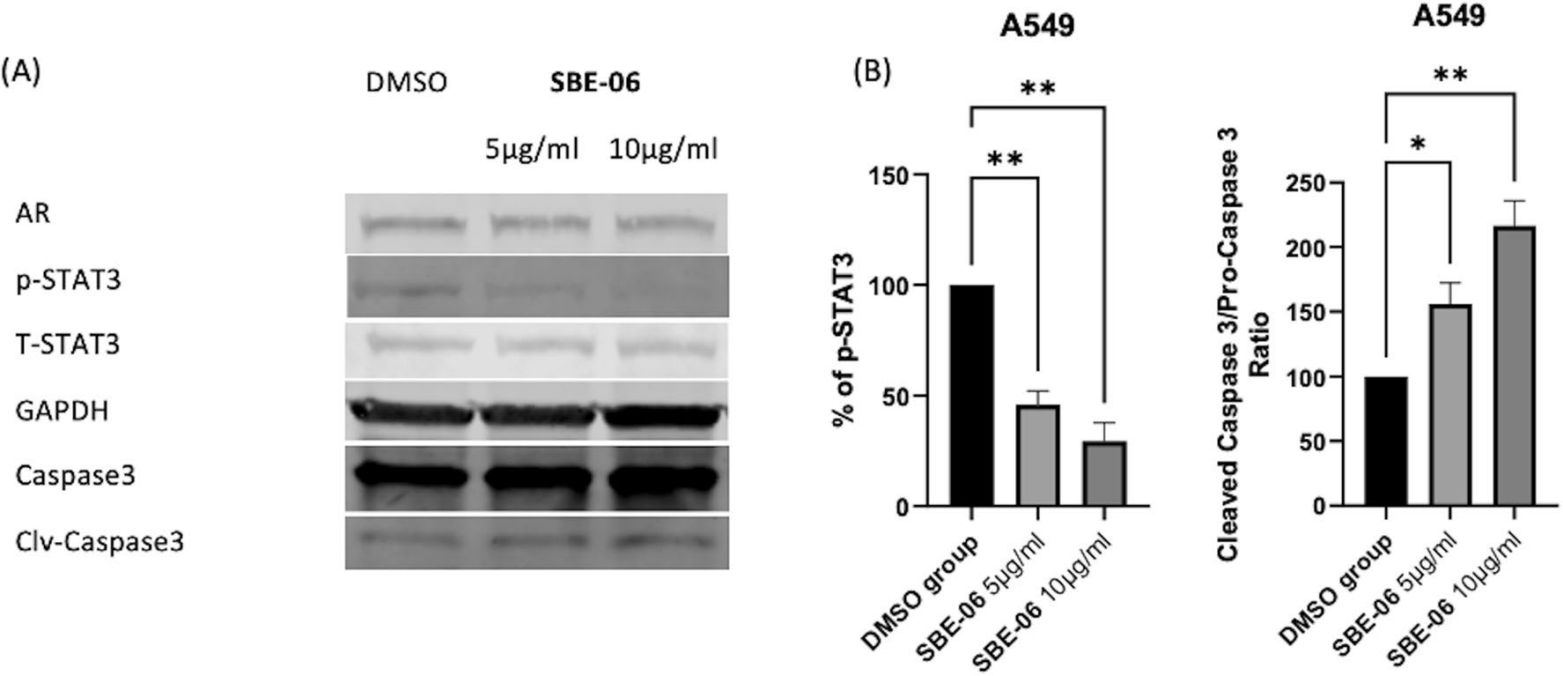
SBE-06 induced apoptosis in A549 cell line via downregulation of p-STAT3 and activation of caspase 3. (**A**) The immunoblot of AR, p-STAT3, T-STAT3, pro-caspase 3, and cleaved caspase 3 in the A549 cell line treated with 0.1% DMSO or 0.1% DMSO solution of SBE-06 (5 and 10 μg/mL) for 24 h. (**B**) Quantification of immunoblot obtained for p-STAT3 and caspase, averaging data from three independent experiments. (Bars show mean plus standard deviation; *p < 0.05; **p < 0.0021).

Intrigued by the enhanced potency of SBE-05, SBE-06 and SBE-07 against A549 cells, we probed further for the effect of SBE-06 on the apoptotic status of A549 cells. We used Western blotting to analyse the effect of SBE-06 on the Signal transducers and activators of transcription 3 (STAT-3) signalling, caspase-3 activation and AR expression^74,75^. We used GAPDH to control for protein loading. While SBE-06 does not affect the levels of AR in this cell line, it resisted the STAT3 activation via downregulation of p-STAT3 and caused the activation of caspase-3 through induction pro-caspase-3 cleavage (Fig. 4A,B). Collectively, this data suggests that SBE-06 induced apoptosis in the A549 cell line.

### In vivo studies: evaluation of the protective effect of apigeninidin-rich SBE on the hepatorenal system of AFB1-challenged rats

**Figure 5:**
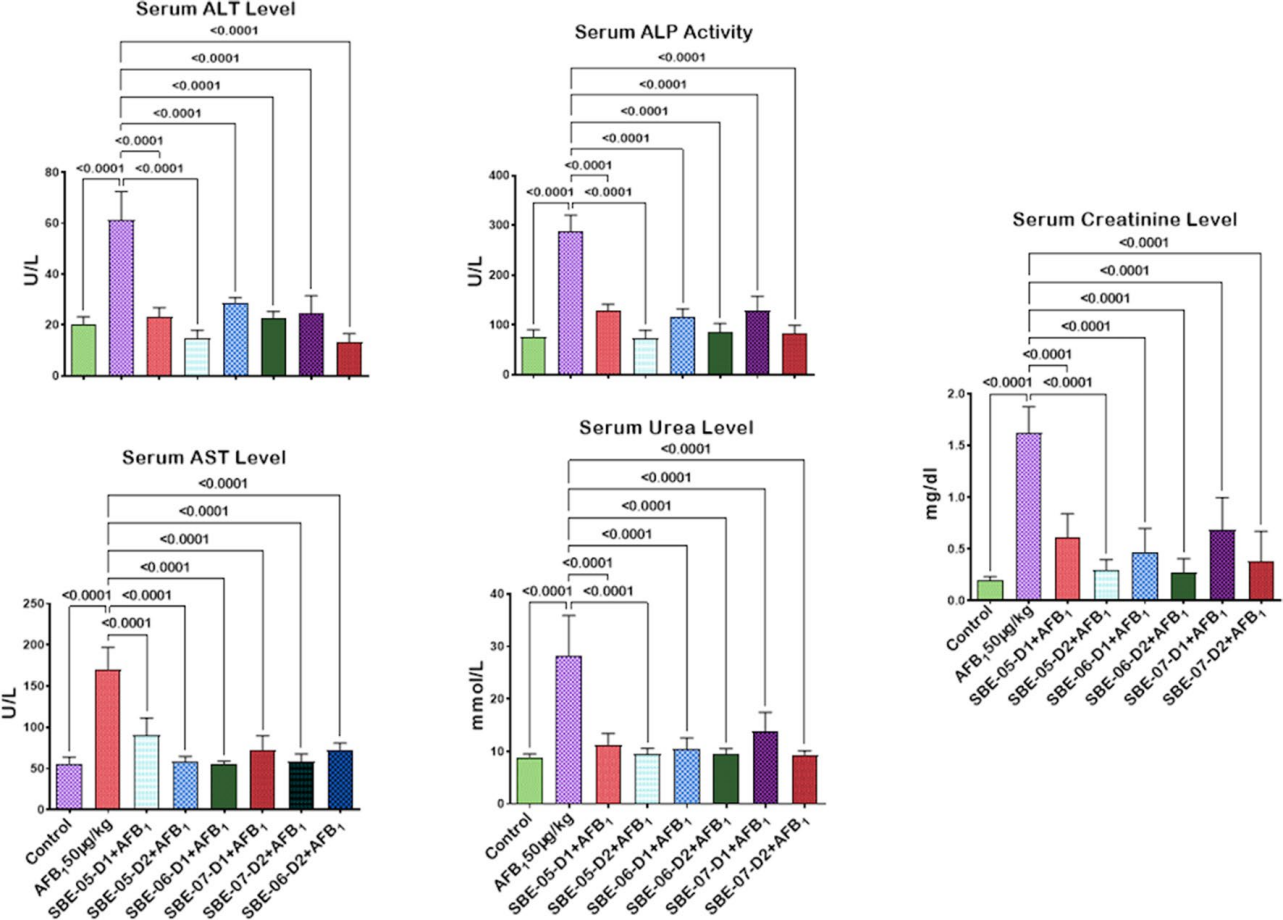
Effect of SBE-05, SBE-06 and SBE-07 on the liver and kidney function of rats treated with AFB_1_ for 28 d. Experimental doses: AFB_1_ at 50 μg/kg; SBE-05-D1 at 5 mg/kg; SBE-05-D2 at 10 mg/kg; SBE-06-D1 at 5 mg/kg; SBE-06-D2 at 10 mg/kg; SBE-07-D1 at 5 mg/kg; SBE-07-D2 at 10 mg/kg. Values are expressed as mean ± SD for 6 rats per treatment cohorts. Connecting lines indicate groups compared to one another, the significance level was set at *(p* < *0.05)*; *p < 0.05; **p < 0.01; *** *p* < 0.001; ****p < 0.0001: indicates the level of significance; *p* > 0.05: not significant. *AFB*_*1*_ Aflatoxin B1; *D1* lower dose; *D2* higher dose; *ALT* Alanine amino transferase; *AST* aspartate amino transferase, *ALP* Alkaline phosphatase; *GGT* gamma-glutamyl transferase.

**Figure 6:**
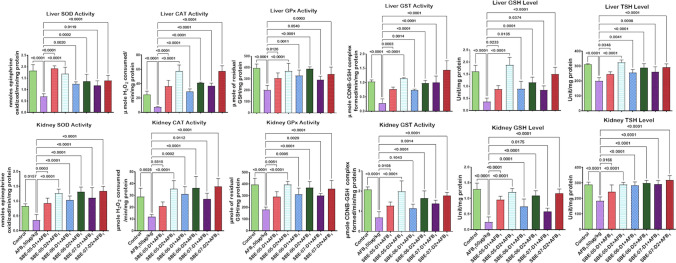
Effect of SBE-05, SBE-06 and SBE-07 on the tissue concentrations of CAT, SOD, GPx, GST, GSH and TSH in the liver and kidney of rats treated with AFB_1_ for 28 d. Experimental doses: AFB_1_ at 50 μg/kg; SBE-05-D1 at 5 mg/kg; SBE-05-D2 at 10 mg/kg; SBE-06-D1 at 5 mg/kg; SBE-06-D2 at 10 mg/kg; SBE-07-D1 at 5 mg/kg; SBE-07-D2 at 10 mg/kg. Values are expressed as mean ± SD for 6 rats per treatment cohorts. Connecting lines indicate groups compared to one another, the significance level was set at *(p* < *0.05)*; *p < 0.05; **p < 0.01; ***p < 0.001; ****p < 0.0001: indicates the level of significance; *p* > 0.05: not significant. *AFB*_*1*_ Aflatoxin B1; *D1* lower dose; *D2* higher dose; *SOD* Superoxide dismutase; *CAT* Catalase; *GPx* Glutathione peroxidase; *GST* Glutathione S-transferase; *GSH* reduced glutathione; *TSH* Total sulfhydryl group.

**Figure 7:**
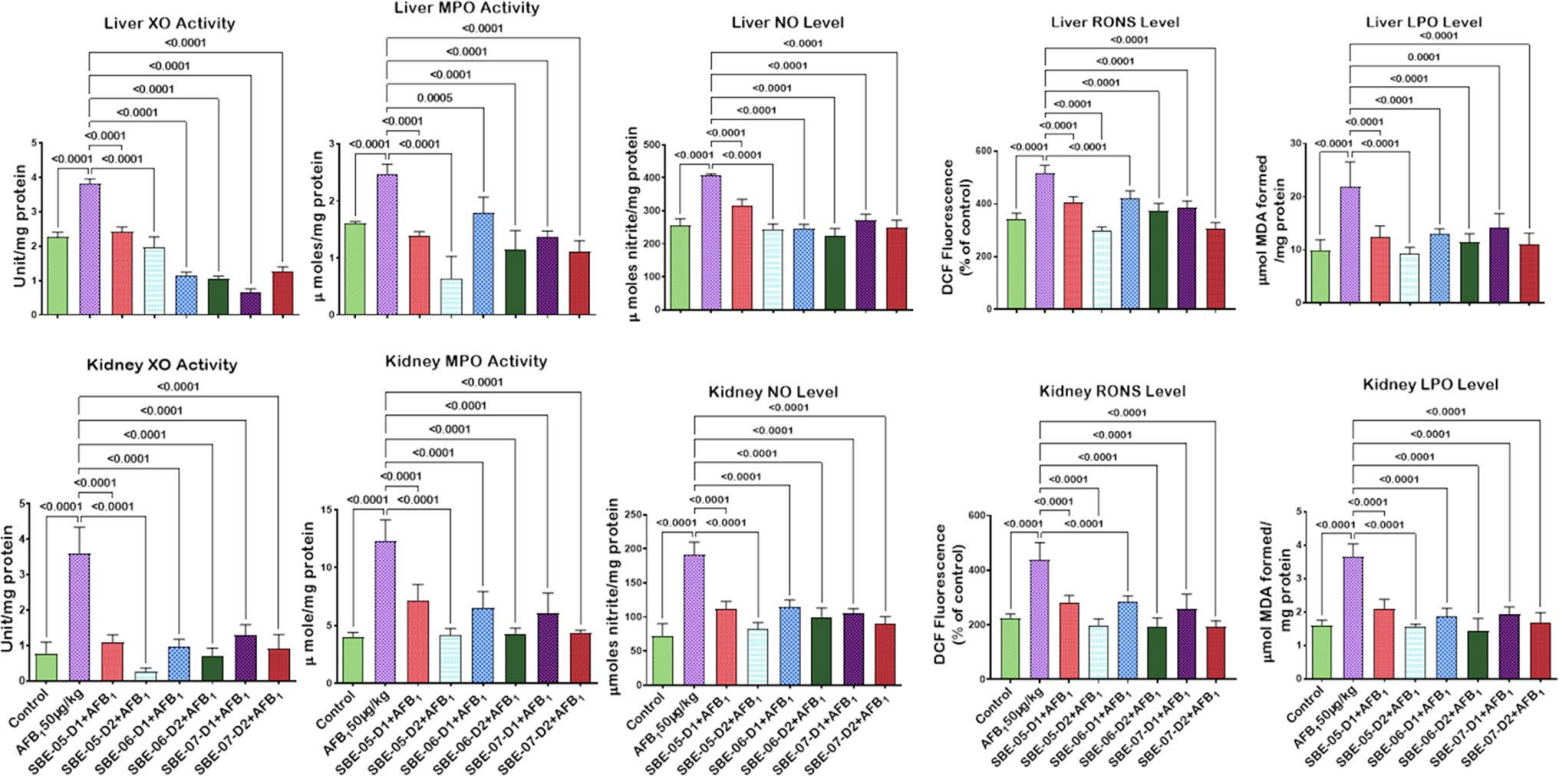
Effect of SBE-05, SBE-06 and SBE-07 on the level XO, LP, RONS, NO, and MPO in the liver and kidney of rats treated with AFB_1_ for 28 d. Experimental doses: AFB_1_ at 50 μg/kg; SBE-05-D1 at 5 mg/kg; SBE-05-D2 at 10 mg/kg; SBE-06-D1 at 5 mg/kg; SBE-06-D2 at 10 mg/kg; SBE-07-D1 at 5 mg/kg; SBE-07-D2 at 10 mg/kg. Values are expressed as mean ± SD for 6 rats per treatment cohorts. Connecting lines indicate groups compared to one another, the significance level was set at *(p* < *0.05)*; *p < 0.05; **p < 0.01; ***p < 0.001; ****p < 0.0001: indicates the level of significance; *p* > 0.05: not significant. *AFB*_*1*_ Aflatoxin B1; *D1* lower dose; *D2* higher dose; *XO* Xanthine oxidase; *LPO* Lipid peroxidation; *RONS* Reactive oxygen and nitrogen species; *NO* Nitric oxide; *MPO* Myeloperoxidase.

**Figure 8:**
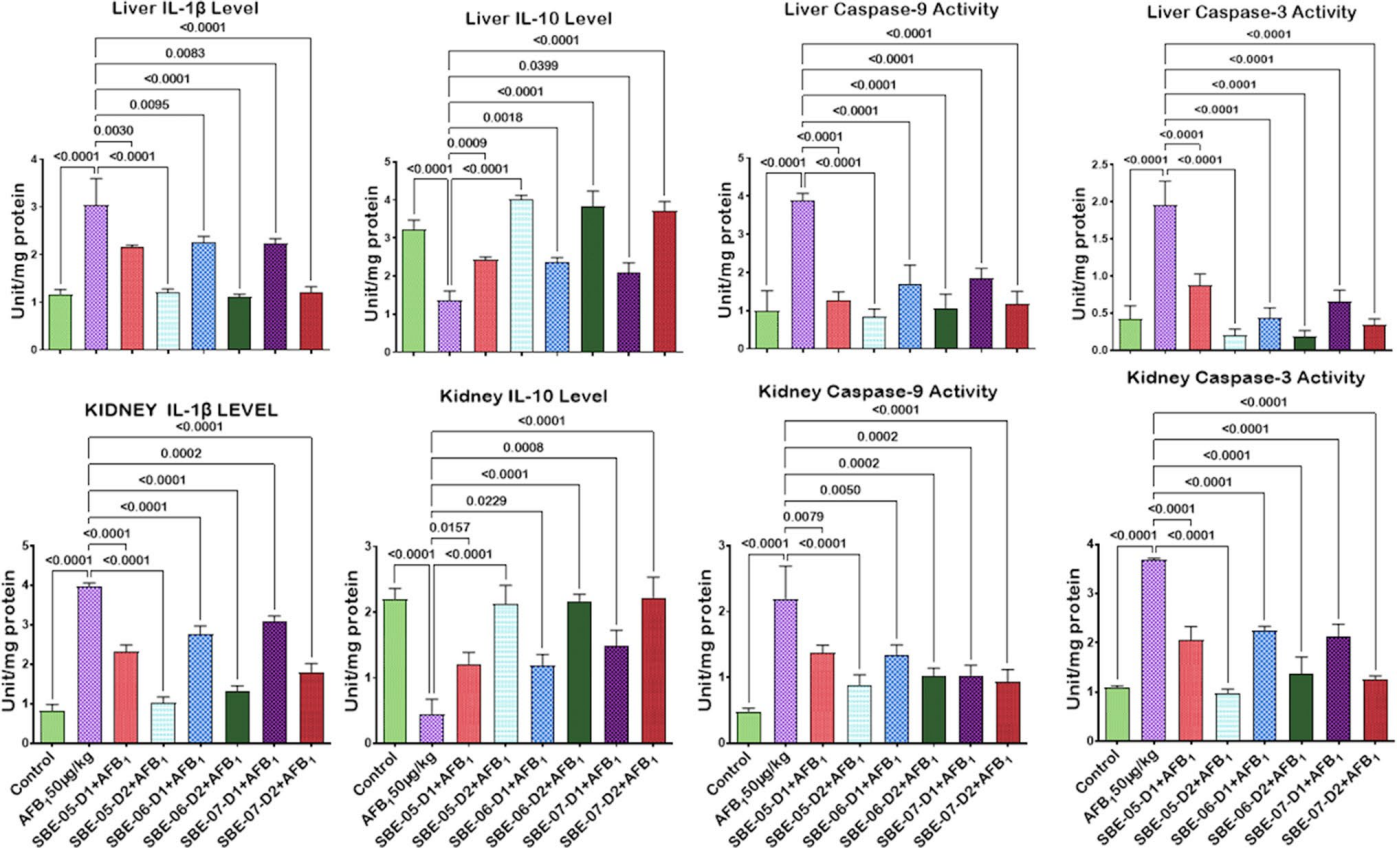
Effect of SBE-05, SBE-06 and SBE-07 on the levels of IL-1β, IL-10, caspase 9 and caspase 3 in the liver and kidney of rats treated with AFB_1_ for 28 d. Experimental doses: AFB_1_ at 50 μg/kg; SBE-05-D1 at 5 mg/kg; SBE-05-D2 at 10 mg/kg; SBE-06-D1 at 5 mg/kg; SBE-06-D2 at 10 mg/kg; SBE-07-D1 at 5 mg/kg; SBE-07-D2 at 10 mg/kg. Values are expressed as mean ± SD for 6 rats per treatment cohorts. Connecting lines indicate groups compared to one another, the significance level was set at *(p* < *0.05)*; *p < 0.05; **p < 0.01; ***p < 0.001; ****p < 0.0001: indicates the level of significance; *p* > 0.05: not significant. *AFB*_*1*_ Aflatoxin B1; *D1* lower dose; *D2* higher dose; *IL-1β* Interleukin-1beta; *IL-10* Interleukin-10.

**Figure 9:**
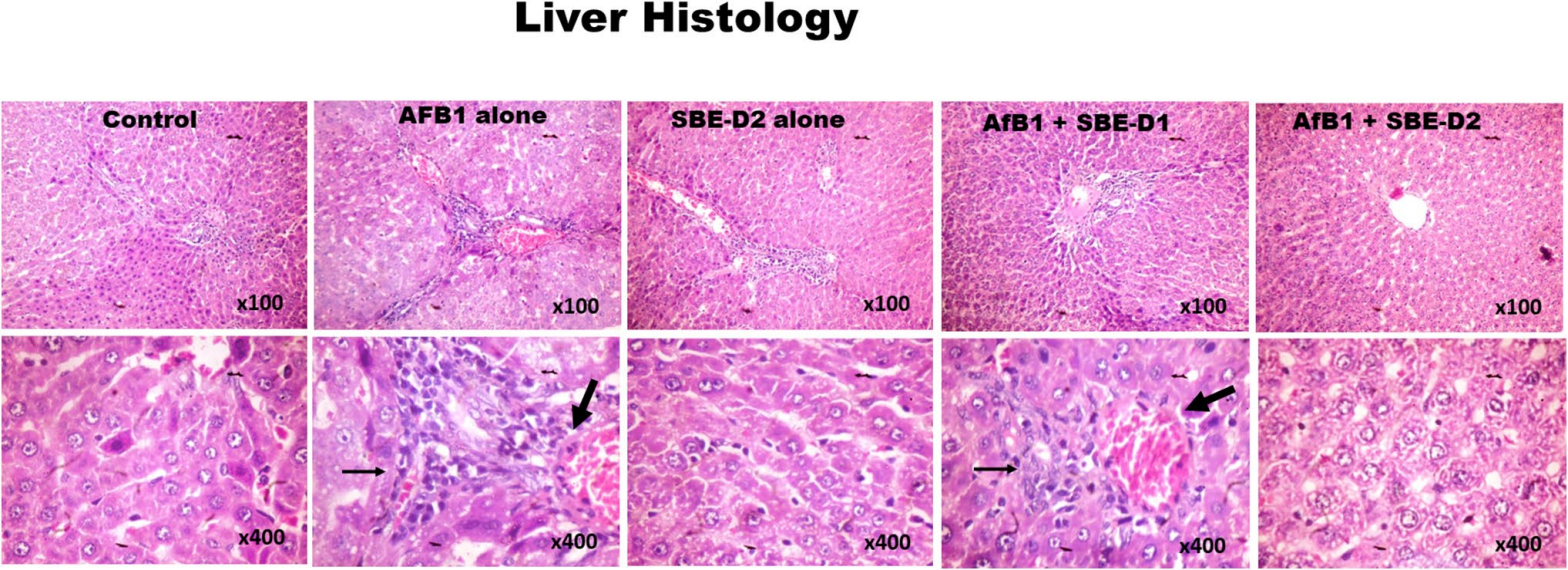
Control Plates of the liver show a focal area of mild congestion and apoptotic bodies with typical tissue architecture. AFB_1_ alone show areas of focal congestion (bold arrows), infiltration of zone 2 by inflammatory cells, mild hydropic/ballooning degeneration of the hepatocytes and moderate microvesicular steatosis (tiny arrows). SBE-D2 alone plate tissue morphologies are like those from control. AFB_1_ with SBE-D1 and SBE-D2 plates improved hepatic cyto-architecture with mild focal congestion and infiltration of zone 2 by inflammatory cells. H and E-stained sections; Magnification at × 100 top panel; × 400 lower panel.

**Figure 10:**
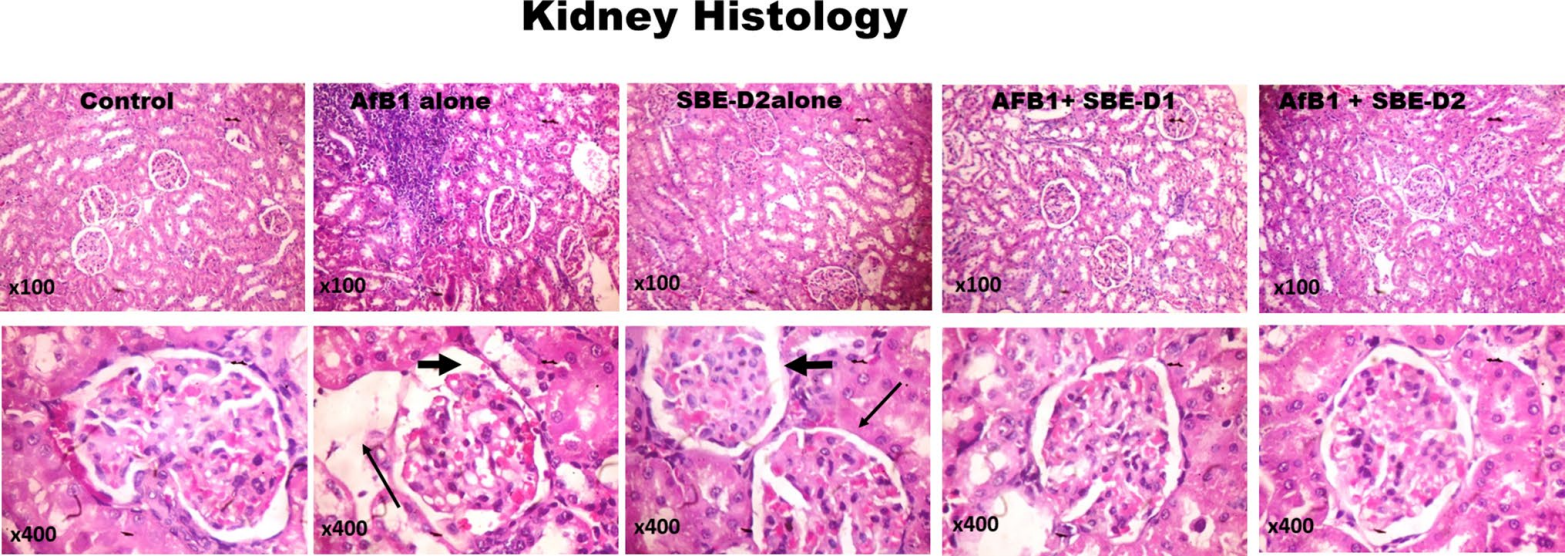
Control plates of the kidney; shows typical architectural features of the kidney tubules devoid of visible lesions. AFB_1_ alone plates show disseminated glomerular messangialisation (thin arrow) and extensive area of infiltration of the cortex by inflammatory cells (bold arrowhead). SBE-D2 plate tissues appear normal and relatively like those from control tissue sections. AFB_1_ with SBE-D1 and SBE-D2 plates dose-dependently improved histo-architecture of the kidney with the mild presence of the inflammatory cell. H and E-stained sections; Magnification at × 100 top panel; × 400 lower panel.

Given that *S. bicolor* is less susceptible to aflatoxin contamination in the field^37,38^, we next probed further for the effects of SBE-05, SBE-06 and SBE-07 on the hepatorenal system of male Wistar rats challenged with AFB_1_. Specifically, we measured the effect of SBEs on organosomatic indices of rats, including mean body weights, organ weights and relative organ weights of the liver and kidney. Compared to the mean body weight before treatment, animals' final mean body weights increased significantly (*p* < 0.05) in the control, AFB_1_, and AFB_1_ + SBE-07-D_1_ experimental cohorts, but non-significantly in other groups. In addition, the mean weight gain of the AFB_1_ + SBE-05-D_1_ cohort was less than other experimental cohorts (Table 3). Since this measurement is not an index of toxicity, we went further to measure the effects of SBE-05, SBE-06 and SBE-07 on the hepatorenal functional parameters. We observed that SBE-05, SBE-06 and SBE-07 at 5 and 10 mg/kg decreased significantly (*p* < 0.05) the increase in the activities of AST, ALT, and ALP as well as levels of urea and creatinine, in the sera of rats caused by exposure to AFB_1_. These extracts reduced these hepatorenal indices' activities and levels to quantities comparable to the control cohort's (Fig. 5). Encouraged by this observation, we further investigated the capacity of SBE-05, SBE-06 and SBE-07 to alleviate oxidative stress that results from the exposure of rats AFB_1_. We observed that SBE-05, SBE-06 and SBE-07, at 5 and 10 mg/kg, reversed the AFB_1_-induced alterations in antioxidant-ROS balance in the liver and kidney of rats to levels comparable to that of the control cohorts. Specifically, relative to the AFB_1_-cohort, these extracts increased the activities and levels of SOD, CAT, GST, GPx, GSH, and TSH (*p* < 0.p05) (Fig. 6) while significantly lowering (*p* < 0.05) those of XO, MPO, NO, RONS, and LPO (MDA) (Fig. 7). Observing that these extracts could avert AFB_1_-mediated oxidative and nitrosative stress in rats, we further explored their anti-inflammatory and antiapoptotic potentials using the ELISA method. We observed that these extracts decreased significantly (*p* < 0.05) the concentration of pro-inflammatory cytokine (IL-1β), and activities of caspase-9 and -3, markers of apoptosis (*p* < 0.05) but significantly lowered the concentration of anti-inflammatory cytokine (IL-10) in the liver and kidney of rats (Fig. 8). Finally, the histological examination of liver and kidney sections revealed that SBE-05, SBE-06 and SBE-07 preserved the histoarchitectural structures of the liver (Fig. 9) and kidney (Fig. 10) similar to that of the control animals.

**Table 3:** Body weight gain and relative Liver and Kidney weight of rats following exposure to AFB1 for 28 days.

	Control	AFB1	SBE-05-D1 + AFB1	SBE-05-D2 + AFB1	SBE-06-D1 + AFB1	SBE-06-D2 + AFB1	SBE-07-D1 + AFB1	SBE-07-D2 + AFB1
Initial body weight (g)	164 ± 21.41	158.50 ± 14.71	161.17 ± 16.12	162.83 ± 11.79	162.67 ± 17.80	162.33 ± 11.69	164.50 ± 11.78	156.66 ± 65.53
Final body weight (g)	229.40 ± 21.87*	220.67 ± 14.14*	190.17 ± 23.54^ ns^	217.00 ± 15.30^ ns^	218.50 ± 18.37^ ns^	229.20 ± 22.37^ ns^	235.00 ± 19.34*	211.33 ± 15.78^ ns^
Weight change (g)	65.40 ± 6.80	62.17 ± 9.41^ ns^	29.00 ± 19.15*	51.50 ± 24.69^ ns^	55.83 ± 14.78^ ns^	65.16 ± 16.29^ ns^	70.50 ± 10.88^ ns^	54.67 ± 12.82^ ns^
Liver weight (g)	6.47 ± 0.80	5.98 ± 0.43^ ns^	5.50 ± 0.75^ ns^	5.73 ± 0.57^ ns^	6.23 ± 0.51^ ns^	6.44 ± 1.41^ ns^	6.47 ± 0.46^ ns^	5.85 ± 0.37^ ns^
Kidney weight (g)	1.16 ± 0.15	1.15 ± 0.08^ ns^	1.12 ± 0.13^ ns^	0.98 ± 0.13^ ns^	1.05 ± 0.08^ ns^	1.18 ± 0.08^ ns^	1.23 ± 0.18^ ns^	1.11 ± 0.04^ ns^
Relative Liver weight (%)	2.86 ± 0.35	2.72 ± 0.23^ ns^	2.90 ± 0.31^ ns^	2.64 ± 0.18^ ns^	2.86 ± 0.13^ ns^	2.86 ± 0.74^ ns^	2.77 ± 0.33^ ns^	2.79 ± 0.34^ ns^
Relative Kidney weight (%)	0.51 ± 0.10	0.52 ± 0.04^ ns^	0.60 ± 0.16^ ns^	0.45 ± 0.07^ ns^	0.48 ± 0.06^ ns^	0.53 ± 0.09^ ns^	0.53 ± 0.07^ ns^	0.53 ± 0.05^ ns^

AFB (50 µg/kg); SBE-05-D1, SBE-05-D2, SBE-06-D1, SBE-06-D2, SBE-07-D1 and SBE-07-D2 (10 and 20 mg/Kg respectively) body weight; n = 6. Data are expressed as mean ± SD. ^ns^: *p* > 0.05 versus Control; ^ns^: *p* > 0.05 versus AFB1 alone (using ANOVA); *: *p* < 0.05 versus FBW; ^ns^: *p* < 0.05 versus FBW (using paired t-test). *AFB1* Aflatoxin B1; *SBE* Sorghum bicolor extract, *D1* lower dose; *D2* higher dose.

In contrast, the liver and kidney of AFB_1_-challenged rats showed evidence of focal congestion, infiltration of zone 2 by inflammatory cells, mild hydropic/ballooning degeneration of the hepatocytes and moderate microvesicular steatosis. They disseminated glomerular messangialisation and extensive area of infiltration of the cortex by inflammatory cells, respectively. These observations validate our in vitro cytotoxicity study that the apigeninidin-rich SBE-05, SBE-06 and SBE-07 are relatively less toxic to normal cells and also revealed that they could drive the restoration of cellular and tissue homeostasis in AFB_1_-challenged rats.

## Discussion

*S. bicolor* contains several bioactive compounds that are beneficial to health. Some of these compounds have potential as prophylactic and curative agents for managing non-communicable diseases such as diabetes mellitus^76^, obesity^77,78^, dyslipidemia^78^, cancer^79,80^, cardiovascular disease^81^, and anaemia^82^. Several *S. bicolor* bioactive compounds have been isolated, and their structures elucidated using NMR and mass spectrometry techniques. These compounds include phenolic acids, tannins, flavonoids, stilbenes, policosanols, phytosterols, 3-deoxyanthocyanidins, proanthocyanidins and flavan-4-ols^34^. Among these phytochemicals, 3-deoxyanthocyaninidins have attracted much attention for their application in the food industry and health, with 56 publications indexed in PubMed between 1987 and 2021. 3-deoxyanthocyanidins comprises apigeninidin, apigeninidin-5-glucoside, luteolinidin, luteolinidin-5-glucoside reported to be abundant in varieties of *S. bicolor*^83–85^.

To further bridge the knowledge gap on the benefits of *S. bicolor* in ethnomedicine*,* we investigated the in vitro anticancer effects of different fractions (SBE05, BSE06, and SBE07) obtained from *S. bicolor* sheath. Moreover, we probed the abrogative effect of these extracts on AFB_1_-mediated liver and kidney damage in rats. Our LC–MS analysis revealed that these *S. bicolor* sheath extracts are replete in apigeninidin, a 3-deoxyanthocyanidin widely utilised in the food industry as natural food colourant^86,87^. When screened against selected cell lines—VERO, Hep-G2, A549, MDA-MB-231, MCF-7, LnCaP and DU145—we observed these fractions had enhanced cytotoxicity against A549 in the order SBE-05 < SBE-07 < SBE-06 but were less toxic to the VERO cell. To the best of our knowledge, this is the first report of apigeninidin-rich *S. bicolor* extracts that elicit selectivity for a lung adenocarcinoma cell line (A549). Previous reports had reported anticancer effects against a wide range of cancer cells, including leukaemia (HL-60)^73^, breast cancer (MCF-7 and MDA-MB 231)^80,88^, cancer cell of the colon (HT-29) and liver (HepG2)^89,90^, malignant cells of colonocytes^91^ and hepatoma (Hepa1c1c7)^92^.

Furthermore, our current findings and previous studies agree with epidemiological studies, which indicate that increased intake of *S. bicolor* is linked to reduced risk of certain types of cancers^93^. To elucidate the mechanism(s) of the anticancer activity of these extracts, we probed the effects of SBE-06, the most potent of the extracts, on the status of selected markers of proliferation and apoptosis in A549 cells. We observed that SBE-06 inhibited the activation of STAT3 via downregulation of phosphorylated STAT3 (p-STAT3) and orchestrated the activation of caspase 3 through induction of the cleavage of cleavage pro-caspase-3. These data show that SBE-06 induced apoptosis and suppressed the proliferation in the A549 cell line by inhibiting the phosphorylation of STAT3 and promoting the activation of pro-caspase-3 (Fig. 3 & |Scheme 1). Decreased STAT3 expression retards the generation of pro-inflammatory cytokines needed to drive cancer growth, invasion, and metastasis, while increased levels of activated caspase-3 will commit the A549 cells to apoptosis. This observation agrees with previous findings, which revealed that extracts of red sorghum bran rich in 3-deoxyanthocyanidin induce apoptosis through the activation of group II proapoptotic proteins such as Bak and Bax, mediate the release of mitochondrial cytochrome C and apoptosis-inducing factor (PIF) into the cytoplasm and derepressing of caspase-9 and caspase-3 in HL-60 cell line^94^. Additionally, extracts of sorghum enriched in phenolic compounds have been shown to inhibit STAT3 phosphorylation in MDA-MB-231 and MCF-7 cell lines^80^.

Since *S. bicolor* sheaths contain active principles with antioxidant, anti-inflammatory and chemo-preventive activities, we hypothesise that our extracts are more likely to alleviate the toxic effects of AFB_1_ in the rat model. We considered the AFB_1_ model of toxicity because it has been reported that AFB_1_ exposure orchestrates hepatocellular carcinoma^95^ and lung adenocarcinoma in humans^96^, thus making it a public health issue globally^97,98^. To this end, we co-treated adult male Wistar Albino rats with different doses of AFB_1_ and SBE-05, SBE-06 and SBE-07. We probed for the effects of this treatment on the levels and activities of crucial hepatic and kidney enzymes in the test animals’ sera. The dosages of the extracts that we investigated (5 and 10 mg/kg) are much lower than the concentration of the extracts that induced toxicity to the VERO cells (equivalent to 60 mg/kg). The enzymes we investigated are standard markers of hepatic and renal functions. Specifically, hepatic enzymes ALT and AST are localised in the cytosol while ALP is housed in the mitochondria of hepatocytes and hence are not exuded into the blood. However, these enzymes percolate in hepatocyte damage into the blood, indicating hepatic injury. Urea and creatinine are important biomarkers of renal function and are produced from creatinine phosphate in the muscles and the breakdown of protein, respectively. In a healthy kidney, these by-products are easily removed from the urine by the kidney, but following kidney injuries, they accumulate in the blood, an indication of renal damage^14,63^. We observed that these apigeninidin-rich extracts resisted AFB_1_-mediated derangement in the hepatorenal system of male rats. Specifically, our results showed that these extracts prevented abnormal weight change decreased hepatic and renal dysfunction parameters, including ALT, AST, ALP, GGT, urea and creatinine after 28 d exposure to AFB_1_. This observation is similar to the previous report, which revealed that dye from *sorghum* bicolor leaf sheath prevented hepatic damage and the induction of oxidative stress in cisplatin-treated rats^99^. To the best of our knowledge, there is no evidence in the pharmacopoeia depicting the protective effect of *S. bicolor* bioactive compounds in the kidney of rats exposed to toxic agents such as AFB_1_.

Oxidative stress, inflammation and apoptosis are hallmarks of several chronic human diseases and toxic chemicals that orchestrate diseases in humans and animals by disrupting the tight communications between redox, anti/pro-inflammatory and anti/pro-apoptotic signalling different tissues. This study validated earlier observations that AFB_1_ triggers RONS generation increase pro-inflammatory cytokines and caspases activity, thereby predisposing rats to hepatorenal damage. These observations agree with the previous findings^14,20^. These effects may have been brought about by AFB_1_ capability to drive mRNAs' expression of mRNAs of TNF-α**,** IL-6, IL-1β**,** iNOS**,** COX-2**,** NF-κB and Keap1a^25,26^; suppression of Nrf2 and Ho-1 signalling^27,28^ and expression of mRNAs of GSH-Px, SOD, CAT, and GST^25,29^. AFB_1_ also drive the activation of Bax and Caspase 3 and the expression of mRNAs of FAS, FADD, TRADD and caspase 8^30^. These effects collectively cause oxidative/nitrosative stress, inflammation, and apoptosis in the affected organs.

AFB_1_ is converted to a toxic metabolite, AFBO by, CYP isoforms (Scheme 2). AFBO can be directly bio-transformed by glutathione S-transferase (GST) and other phase-2 enzymes in the liver into AFB_1_-mercapturic acid, collected in the kidney and excreted in the urine. A decrease in the level of GST, especially in the absence of reduced GSH and other total sulfhydryl groups (TSH), will further upturn the AFBO load in the body. AFBO, in this state, drives the expression of XO, whose metabolic action results in the production of reactive oxygen species (ROS), e.g., superoxide anion radical (O_2_^.-^). O_2_^.-^ is immediately converted to hydrogen peroxides (H_2_O_2_) by superoxide dismutase (SOD). Catalase and glutathione peroxidase convert this toxic by-product into molecular water. This natural remediation process is needed to reduce the liver and kidney levels of AFBO and other AFB_1_ metabolites. However, where this remedial process is compromised, the level of these enzymes gradually diminishes with a concomitant elevation in the hydroxyl radical concentrations (OH^.^). This process is regarded as the Fenton and Haber–Weiss reactions, and it occurs in the presence of metallic iron (Fe^2+^/Fe^3+^). The liver and kidneys' myeloperoxidase (MPO) concentrations may increase if H_2_O_2_ is not enzymatically neutralised. And may play a role in forming other toxic free radicals, including hypochlorous acid (HOCl). RNS also similarly increases liver and kidney damage. The biochemical processes that lead to the formation of RNS are chiefly contributed by nitric oxide synthases, including inducible nitric oxide synthase (iNOS), endothelial nitric oxide synthase (eNOS) and neuronal nitric oxide synthase (nNOS)^100^, with the evolution of nitric oxide (NO). NO, in the presence of O_2_^.-^, triggers the generation of peroxyl nitrite (ONOO^-^). The intermediates –O_2_^.-^H_2_O_2_, OH^.^, HOCl, NO, and ONOO^—^ -are assayed collectively as indicators of reactive oxygen and nitrogen species (RONS). RONS may interact with cellular biomolecules forming deleterious protein crosslinks, lipid peroxides (malondialdehyde) and DNA adducts in the liver and kidney. Consequently, these adducts will trigger inflammation and apoptosis, harming the hepatorenal system^14,63^. We inferred that these apigeninidin-rich fractions counterbalanced the shifts in redox rheostat as evidenced by the increase in the liver and kidney concentrations of SOD, CAT, GST, GPx, GSH and TSH with a corresponding decrease in XO, MPO, NO, LPO (MDA), and RONS (Scheme 2).

The observed AFB_1_-induced inflammation is ascribed to IL-1β increase and reduced IL-10 in rats' liver and kidneys. Therefore, we infer that the interaction of IL-1β with the IL receptor will result in the activation of MAPK, which in turn activates IKK. IKK is known to mediate the release of NF-kB from NF-kB: IkB complex, which then enters the nucleus and drives the expression of more pro-inflammatory cytokines (Scheme 1). The contribution of IL-1β in the mediation of inflammation and apoptosis has long been established, and current evidence reveals that IL-1β can orchestrate inflammation and apoptosis by stimulating the expression of IL-6, TNF-α, Bax and caspase 3^101^. Our findings show that our apigeninidin-rich extracts resolved AFB_1_-mediated inflammation by lowering the level of IL-1β and increasing IL-10 in the liver and kidney of rats. The anti-inflammatory effect of apigeninidin reported in the current study agrees with previous studies^102^, where apigeninidin was reported to target inflammation through the cyclo-oxygenase-2 (COX-2) and prostaglandin E2 (PGE-2) blockade.

AFB_1_-mediated apoptosis of the liver and kidney is attributed to high caspase 9 and caspase 3—the initiator and executioner of apoptosis, respectively. Based on this observation, it is likely that the increased liver and kidney activities of caspase 9 and caspase 3 may have been brought about by the expression of p53 following alteration in redox balance and unresolved inflammation. The tumour suppressor gene, p53, drives the expression of the p53 upregulated modulator of apoptosis (PUMA). This pro-apoptotic protein modulates the Bax/Bcl-2 ratio in favour of the expression of more Bax^103^. Bax then translocates into the mitochondria and causes the evolution of cytochrome C in a process termed mitochondrial permeability transition pore (MPTP). Cytochrome C egress from the mitochondria into the cytoplasm, where it interacts with apoptotic peptidase activating factor 1 (APAF 1) and pro-caspase 9 to form apoptosome. The formation of this complex is needed to activate caspase 9, which then cleaves pro-caspase 3 into caspase 3—the executioner of programmed cell death as observed in the liver and kidney tissues (Scheme 2) as evidenced in our study (Fig. 7). AFB_1_-mediated apoptosis was averted by our apigeninidin-rich extracts as revealed by a decrease in the tissue levels of caspase 9 and caspase 3. These findings corroborate a previous study from our laboratory in which we reported that antioxidant gallic acid prevented liver and kidney damage in AFB_1_ challenged rats^14^.

Histoarchitectural evidence is vital for assessing the degree of toxicity impacted on the tissues of animals following exposure to toxic chemicals. It forms the basis for most clinical generalisations after diagnosis^104,105^. It becomes practically impossible to substantiate biochemical and molecular-based claims in clinical and experimental findings without histological evidence. In our current study, AFB_1_ disrupted the histoarchitectural frameworks of the liver and kidney tissues. Injury to the hepatorenal tissue is recognised as a hallmark of excessive RONS. These molecules drive the infiltration of monocytes from the blood into AFB_1_-perturbed tissues, where they differentiate to Kupffer cells and intraglomerular mesangial cells^106,107^. These pro-inflammatory cells interact with AFB_1_-mediated danger-associated molecular patterns (DAMPs) through their pattern recognition receptors (PRRs), notably toll-like receptors and drive the activation of NF-κB and interferon regulatory factor (IRF). These transcription factors ingress the nucleus and orchestrate the expression of genes encoding cytokines, notably IL-1β, TNF-α and IFN-α, -β and other soluble mediators. These soluble mediators activate apoptotic or necrotic signalling pathways leading to the death of liver and kidney tissues^108^. Apigeninidin-enriched SBE fractions at the selected doses inhibited severe necrosis and apoptosis of the liver and kidney by increasing the tissue levels of endogenous antioxidants and anti-inflammatory cytokines to inhibit the generation of ROS and RNS and inflammation needed to drive apoptosis and necrosis. Our findings recapitulate the antioxidant and anti-inflammatory effects of apigeninidin-rich SBE and largely agree with previous studies. For instance, bioactive coumarins such as auraptene, marmin, isoauraptene and meranzinhydrate extracted from *Citrus grandis* peel were reported to inhibit xylene and carrageenan-induced inflammation in vivo^109^ while apigenin, luteolin and fisetin depressed inflammation through the downregulation of TNF-α—induced JNK, ERK, p38, CCL2/MCP-1, and CXCL1/KC activation^110^.

## Conclusion

Taken together, our study shows that apigeninidin-rich extracts from S. bicolor selectively inhibited the growth of A549 cells by downregulating the expression of STAT3 and upregulating the activation of caspase-3. Moreover, these extracts averted the induction of oxidative and nitrosative stress, inflammation, and apoptosis in the hepatorenal system of rats. These observations highlight the potential of these cheap and readily accessible extracts for lung cancer therapy and chemo-preventive agents in preventing aflatoxin-related health issues in developing countries. The shortage of improved storage facilities of agricultural products exposes both human and livestock populations to increase aflatoxin contamination risks. Therefore, we recommend that the apigeninidin-rich *S. bicolor* extracts investigated herein merit extensive preclinical efficacy, toxicology and pharmacokinetic profiling preparatory to their clinical evaluation for managing lung adenocarcinoma and aflatoxicosis in the future.

## Supplementary Information

Supplementary Information.
