# Supplementary material for: Apigeninidin-rich Sorghum bicolor (L. Moench) extracts suppress A549 cells proliferation and ameliorate toxicity of aflatoxin B1-mediated liver and kidney derangement in rats
Source: Sci Rep. 2022 May 6;12:7438. doi: 10.1038/s41598-022-10926-1 (PMC9076626; doi:10.1038/s41598-022-10926-1)

P-STAT3, Caspase3, and Clv-Cas3 (same gel as 2nd page, 800nm)

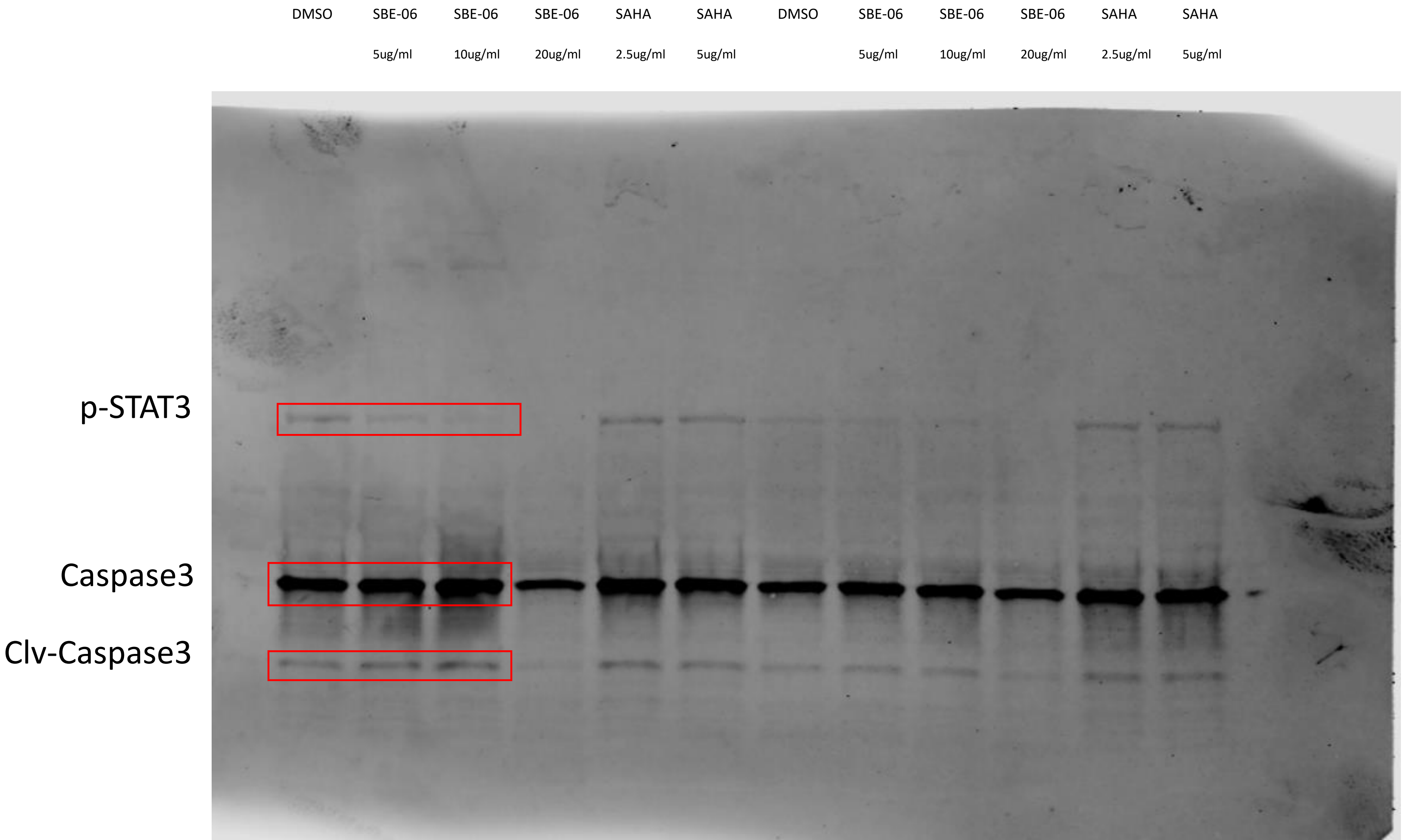

AR and GAPDH (Same gel, wavelength 690nm)

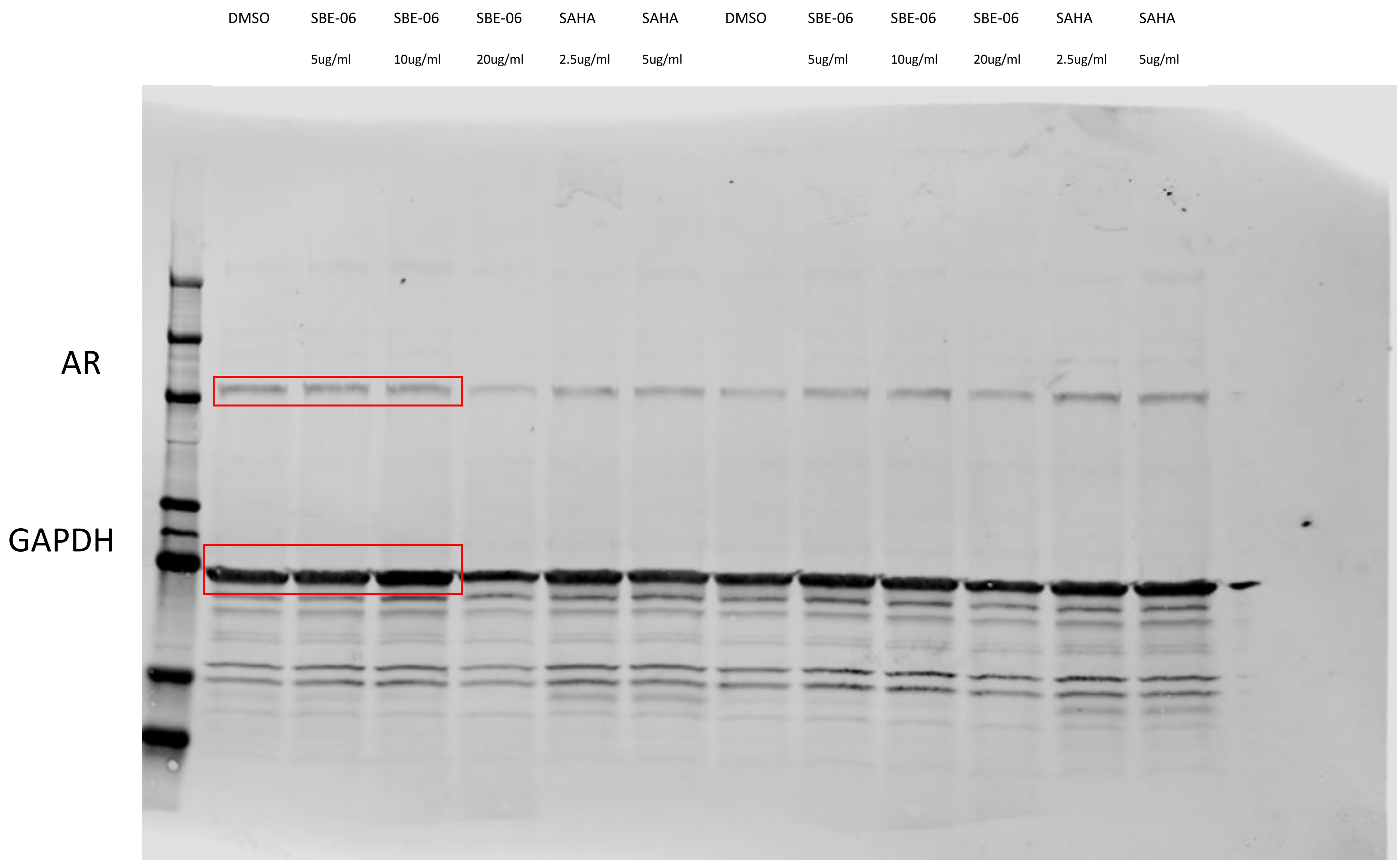

T-STAT3 and GAPDH (2nd gel)

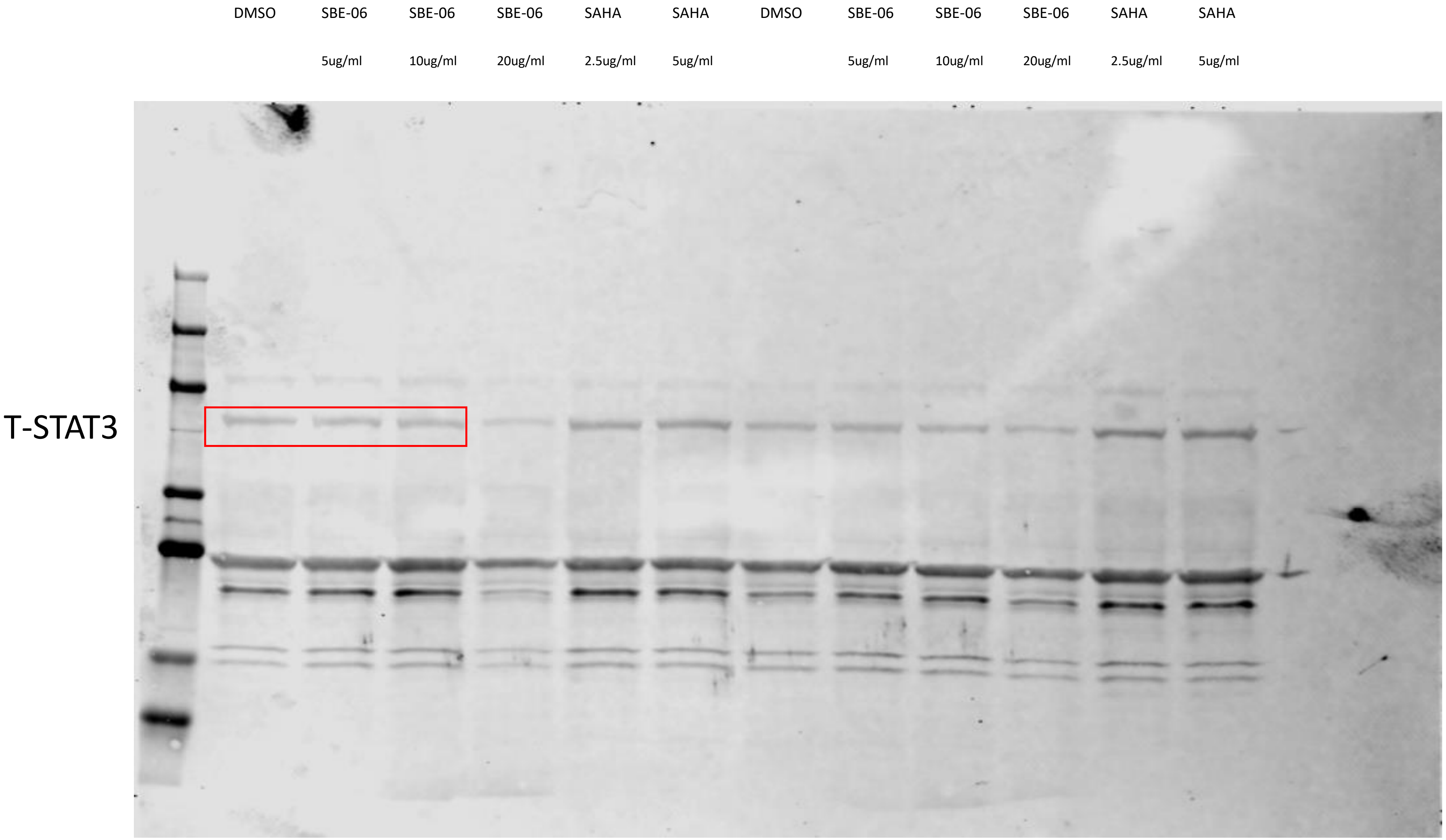

Supplement: Supplementary file 1 — Supplementary Information. [file 41598_2022_10926_MOESM1_ESM.pdf]
